# Nrsn1–Smarcc1 Coupling Regulates Neural Stem Cell Differentiation and Chronic‐phase Recovery After Ischemic Stroke

**DOI:** 10.1002/advs.77547

**Published:** 2026-09-03

**Authors:** Ruolin Zhang, Chang Liu, Yuneng Zhou, Kaichen Zhao, Muyang Li, Bingcheng Cai, Ying Wang, Zhiyuan Yuan, Zhaoxin Liu, Zilong Yuan, Yao Xiao, Peiyang Zhou, Ke Shui, Wendai Bao, Min Zhang, Jun Qin, Jun Chen, Xin Yang, Zhiqiang Dong

**Affiliations:** ^1^ College of Biomedicine and Health College of Life science and Technology Huazhong Agricultural University Wuhan China; ^2^ Hubei Provincial Clinical Research Center for Central Nervous System Repair and Functional Reconstruction Taihe Hospital Hubei University of Medicine Shiyan Hubei China; ^3^ State Key Laboratory of Genome and Multi‐omics Technologies BGI Research Shenzhen China; ^4^ Shanxi Medical University‐BGI Collaborative Center For Future Medicine Shanxi Medical University Taiyuan China; ^5^ Shenzhen Key Laboratory of Single‐Cell Omics BGI Research Shenzhen China; ^6^ Department of Neurology Xiangyang No.1 People's Hospital Hubei University of Medicine Xiangyang Hubei China; ^7^ Hubei Cancer Hospital Tongji Medical College Wuhan Hubei China; ^8^ Department of Electrical and Electronic Engineering School of Engineering Cardiff University Cardiff UK; ^9^ Hubei Jiangxia Laboratory Wuhan Hubei China

**Keywords:** Foxa2, Ischemia‐reperfusion, Neuronal differentiation, Nrsn1, NSC, Smarcc1

## Abstract

Stroke remains a leading cause of long‐term neurological disability worldwide, largely due to irreversible neuronal loss and the limited regenerative capacity of the adult mammalian brain. Neural stem cells (NSCs) in the adult brain possess the potential to generate new neurons after injury, yet the molecular mechanisms regulating their neuronal differentiation following ischemic insult remain incompletely understood. Here, integrating single‐cell multi‐omics analyses with spatial transcriptomics, we systematically delineated cell type‐specific spatiotemporal dynamics in the striatum of a mouse model of ischemia‐reperfusion injury. We identified Neurensin 1 (Nrsn1) as a gene markedly upregulated during NSC‐derived neuronal differentiation in the recovery phase. Mechanistically, Foxa2 directly activates Nrsn1 transcription, whereas Nrsn1 promotes neuronal differentiation by facilitating the nuclear translocation of the chromatin‐remodeling factor Smarcc1 in vitro. In vivo, both endogenous NSCs and transplanted NSCs overexpressing Nrsn1 significantly enhanced neuronal regeneration and improved functional recovery in mice subjected to middle cerebral artery occlusion and reperfusion (MCAO/R). Collectively, these findings identify Nrsn1 as a key regulator of NSC neuronal differentiation and uncover a Nrsn1–Smarcc1 coupling mechanism that promotes neural regeneration after ischemic brain injury, highlighting a potential molecular target for strategies aimed at enhancing post‐stroke recovery.

## Graphical abstract

In the post‐stroke brain, Foxa2 induces the transcriptional upregulation of Nrsn1 in NSCs. Nrsn1 functionally couples with Smarcc1, modulating its nuclear availability and protein abundance, thereby influencing Smarcc1‐associated regulatory programs linked to neuronal lineage commitment. Through this coupling, Nrsn1 promotes the differentiation of NSCs toward neuronal progenitor cells and mature neurons, contributing to neural circuit repair and functional recovery during the chronic phase following ischemic stroke.

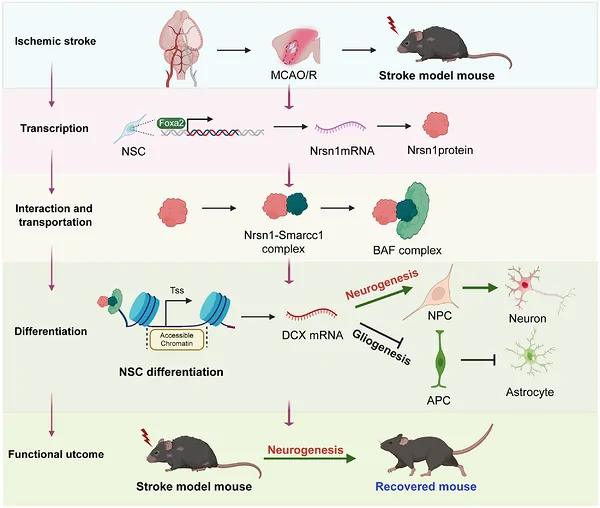

## 1 Introduction

Stroke is consistently ranked as the second leading cause of death across the globe, with ischemic stroke accounting for approximately 80% of all cases [1, 2]. Its pathogenesis involves complex, multicellular damage cascades spanning the cerebral cortex, striatum, and other brain regions. Neuronal loss and apoptosis represent the primary drivers of permanent neurological deficit. Despite advances in acute reperfusion therapies, most patients experience limited functional recovery, owing in part to insufficient blood–brain barrier repair and the failure to reconstruct damaged neural circuits. These challenges underscore the need for novel, mechanism‐based therapeutic strategies.

To address these unmet needs, neural stem cells (NSCs) are promising candidates for stroke therapy due to their capacity to regenerate neurons and rewire damaged neural circuits [3]. Preclinical studies demonstrate that transplanted NSCs differentiate into neurons and improve functional outcomes in ischemic stroke models [4]. However, clinical translation of NSC‐based therapeutic strategy is hindered by two critical bottlenecks, namely poor survival of transplanted NSCs within the post‑ischemic inflammatory microenvironment and inefficient neuronal differentiation, with differentiation rates typically below 5% [5, 6, 7]. Enhancing NSC survival and directing their neuronal lineage commitment are thus rate‐limiting steps for advancing NSC‐based stroke therapy. Our prior work demonstrated that NSC‐derived exosomes combined with NSC transplantation, significantly boost neuronal differentiation and therapeutic efficacy in stroke mice [8]. In addition, existing studies have shown that genetic engineering of NSCs biases their differentiation toward neurons while suppressing glial fate commitment [9].

The striatum, a subcortical region adjacent to the neurogenic subventricular zone (SVZ), is a primary site of ischemic injury and thus represents a key target for post‐stroke recovery. These SVZ neuroblasts migrate to the ischemic boundary zone such as the striatum, where some become spiny projection neurons [10, 11, 12]. This injury‐induced neurogenic response provides an in vivo platform to investigate the molecular regulation of NSC differentiation. Recent advances in single‐cell technologies, such as single‐nucleus RNA sequencing (snRNA‐seq) and single‐nucleus assay for transposase‐accessible chromatin sequencing (snATAC‐seq), allow integrated transcriptomic and epigenomic profiling of NSC activation and fate specification [13, 14, 15]. Spatial transcriptomic approaches such as SpaTial Enhanced REsolution Omics‐sequencing (Stereo‐seq) further preserve anatomical context, allowing precise mapping of NSC migration and differentiation in the heterogeneous post‐ischemic brain [16, 17]. Therefore, dissecting the molecular regulatory mechanisms underlying NSC differentiation in the striatum using high‐throughput sequencing strategies will enable the identification of novel targets for enhancing the reparative potential of both endogenous and transplanted NSCs.

Using the integrated multi‐omics strategy applied to ipsilateral striatum from MCAO/R mice, we identified Neurensin 1 (Nrsn1) as the gene selectively upregulated during NSC‐derived neuronal differentiation. Nrsn1 serves as a critical cell‐intrinsic regulator that governs NSC differentiation into neuronal progenitor cells (NPCs) and mature neurons in the brain post‐stroke. Mechanistically, Foxa2 directly promotes Nrsn1 transcription, and Nrsn1 facilitates neuronal differentiation by enhancing the nuclear translocation of Smarcc1. Furthermore, both Nrsn1‐overexpressing endogenous and transplanted NSCs significantly enhance neural repair and functional recovery. Then, we evaluated the therapeutic potential of Nrsn1‐overexpressing NSCs in MCAO/R mice, with a specific focus on their capacity to restore damaged neural circuits and improve post‐stroke functional recovery. Together, these findings identify Nrsn1 as the previously unrecognized regulator of post‐stroke neurogenesis and provide a mechanistic framework for optimizing NSC‐based regenerative strategies.

## 2 Results

### 2.1 Differentiation of NSCs in the Striatum Post‐Ischemic Stroke

**FIGURE 1:**
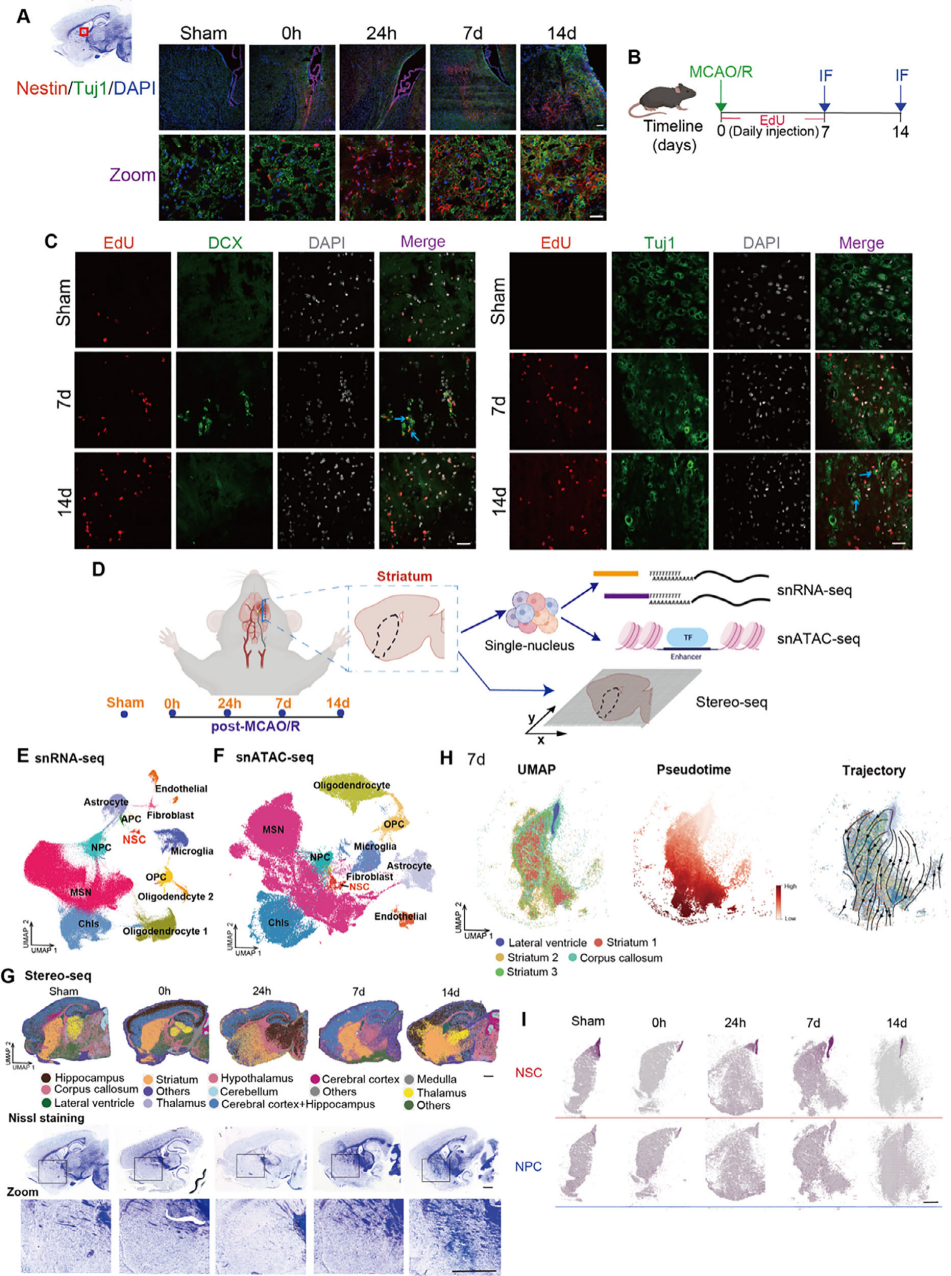
Differentiation of NSCs in the striatum post‐ischemic stroke. (A) Representative IF images of Nestin and Tuj1 in the striatum at different time points post‐stroke. Scale bar: 100 µm. (B) Workflow of the experimental scheme (created in BioRender). (C) IF of EdU, DCX (left) and Tuj1 (right) in the ROI at 7 d and 14 d post‐stroke, and arrow pointing to double positive cells. Scale bar: 100 µm. (D) Workflow of the experimental scheme (created in BioRender). (E) Uniform Manifold Approximation and Projection (UMAP) visualization of cell types identified by snRNA‐seq. (F) UMAP visualization of cell types identified by snATAC‐seq. (G) UMAP visualization of cell types identified by Stereo‐seq. Nissl staining corresponding to Stereo‐seq at different time points post‐stroke. Scale bar: 500 µm. (H) Spatial distribution in the striatum at 7 d post‐stroke (left). spaTrack streamline plots showing the predicted trajectory of cell differentiation in the striatum at 7 d post‐stroke. Areas are colored by pseudotime (middle) and trajectory (right). (I) The spatial visualization of gene modules of NSCs and NPCs by Stereo‐seq at different time points post‐stroke. Scale bar: 500 µm.

To explore dynamics of NSCs during post‐stroke neural repair, we established the MCAO/R mice model. To validate the migration and differentiation of NSCs in the striatum as reported previously [18, 19, 20], we performed immunofluorescence staining (IF) for Nestin (NSC marker) and β‐III tubulin (Tuj1; immature neuron marker) at different time points post‐stroke. Nestin^+^ cells were detected in both the ventral and dorsal striatum at 7 d post‐stroke, whereas Tuj1^+^ cells emerged in the same region at 14 d post‐stroke (Figure 1A; Figure S1A). These findings indicate that NSCs may migrate from the SVZ to the striatum on the seventh day and differentiate into immature neurons by 14 d post‐stroke. To further trace the temporal sequence of NSCs proliferation and differentiation, proliferating cells were labeled with 5‐Ethynyl‐2′‐deoxyuridine (EdU; Figure 1B,C). The results showed that doublecortin (DCX)^+^/EdU^+^ double‐positive cells (representing NPCs) were observed in the striatum at 7 d post‐stroke [21], whereas Tuj1^+^/EdU^+^ cells (representing immature neurons) emerged at 14 d. These data confirm the stepwise progression of NSC‐derived neurogenesis within the post‐ischemic striatum.

Considering the dynamic changes in NSCs following stroke, we collected the ipsilateral striatum from MCAO/R mice at 0 h, 24 h, 7 d, and 14 d post‐reperfusion and Sham‐operated (Figure 1D). snRNA‐seq and snATAC‐seq were subsequently performed on these samples (Figure 1E,F; Tables S1 and S2). For spatial validation, brain sections at matched time points were analyzed using Stereo‐seq (Figure 1G). For snRNA‐seq data and snATAC‐seq, we identified 11 cell types based on canonical cell markers (Figure S1B and Table S3), including NSCs, NPCs, astrocyte precursor cells (APCs), cholinergic interneurons (Chls), medium spiny neurons (MSNs) [22], astrocytes, endothelial cells, fibroblasts, microglia, oligodendrocyte precursor cells (OPCs), and oligodendrocytes.

We then aligned all cell clusters with their corresponding anatomical regions based on the Allen Mouse Brain Atlas [23] to link transcriptional profiles with spatial localization. These clusters displayed regional segregation of brain tissues and were consistent with histological annotations (Figure 1G; Table S4). To further characterize the damaged regions at different time points, brain sections adjacent to the patch implantation site were selected and subjected to Nissl staining (Figure 1G). The neuronal injury was evident in the striatum at 24 h post‐stroke. In the chronic phase (7 d and 14 d), neuronal recovery and Nissl body reformation were observed.

Given the robust single‐cell datasets, we next explored intercellular communication networks to elucidate the regulatory cues for NSC migration. The prior studies have demonstrated that endothelial cells modulate NSCs behavior via vascular endothelial growth factor (VEGF) signaling during tissue homeostasis and regeneration [24, 25], highlighting the need to dissect intercellular crosstalk in NSCs migration [26]. The neuroblasts migrate close to blood vessels through an area exhibiting early vascular remodeling and persistently increased vessel density [20]. Astrocytes also regulate neurogenesis and angiogenesis via secreting neurotrophic and vascular growth factors [27]. We thus detected crosstalk among NSCs, endothelial cells and astrocytes at different time points post‐stroke (Figure S1C). The results showed that the interaction between NSCs and endothelial cells was significantly enhanced post‐stroke, with prominent crosstalk observed at 24 h and sustained up to 14 d, suggesting that endothelial cells may recruit NSCs to migrate toward the striatum. Since adult NSCs persist in the SVZ of lateral ventricles [28], we performed spaTrack analyses [29] based on data from striatum at 7 d post‐stroke to verify NSC migration (Figure 1H). At 7 days post‐stroke, cells marked by the NPC‐specific gene module were widely distributed throughout the striatum, providing additional evidence that NSCs possess the potential to differentiate along the NPC lineage (Figure 1I). These results support the differentiation tendency of cells from the SVZ region to the striatum. Quantitative analysis showed that the number of NSCs increased at 7 d post‐stroke with a subsequent reduction at 14 d, suggesting that NSCs migrate to the striatum and initiate differentiation around 7 d post‐stroke (Figure S1D,E). Collectively, these results confirm that after stroke, NSCs migrate from the SVZ to the striatum and differentiate into immature neurons.

### 2.2 Nrsn1 Acts as a Critical Regulator Governing Neuronal Differentiation of NSCs

**FIGURE 2:**
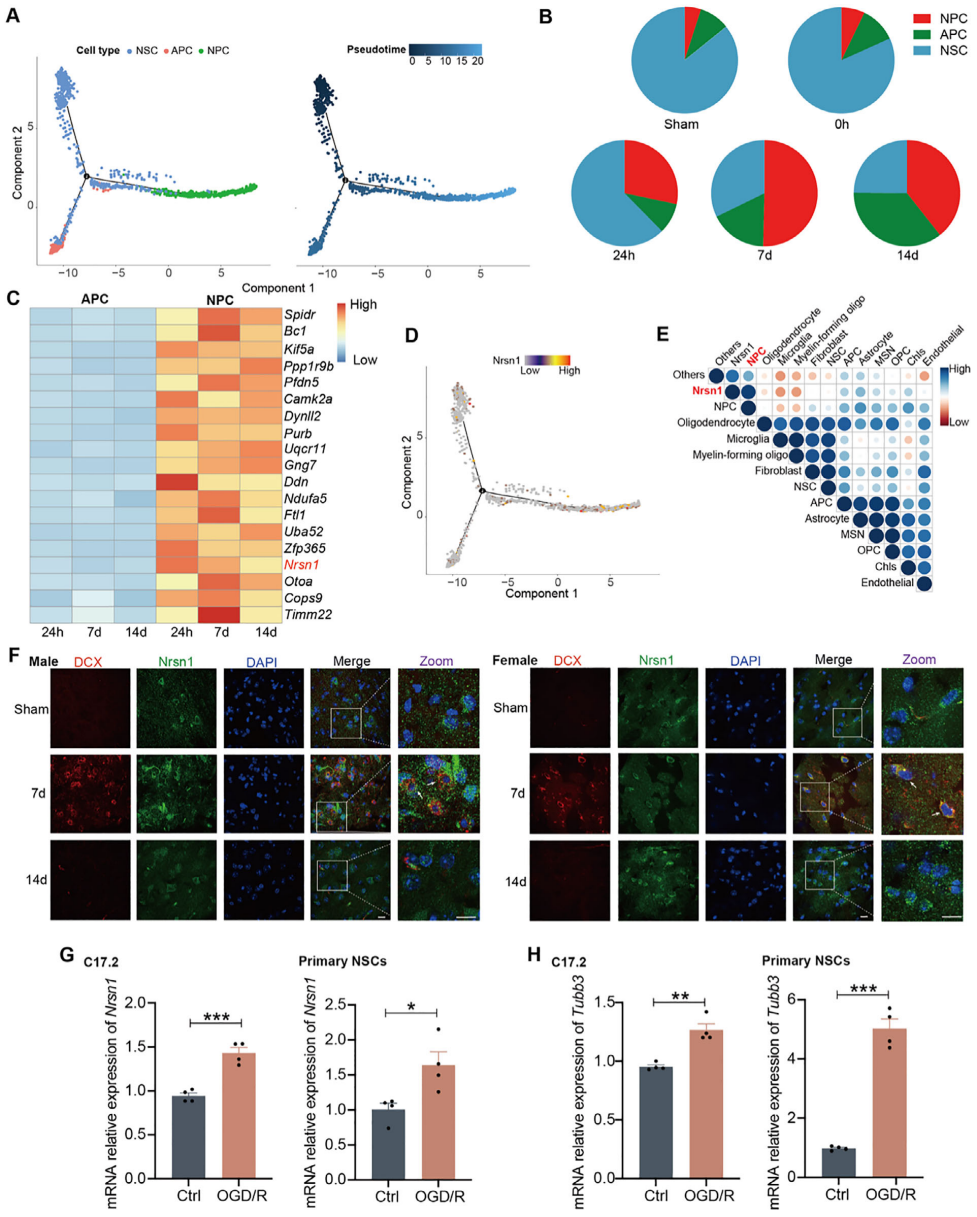
Nrsn1 acts as a critical regulator governing neuronal differentiation of NSCs. (A) Pseudotemporal trajectory analysis of NSCs and their progeny. The left side illustrates the distribution of NSCs (blue), APCs (red), and NPCs (green) along the differentiation trajectory, and the right side is the pseudotime progression, with cells colored from early (light blue) to late (dark blue), demonstrating the bifurcation of NSC differentiation into the astrocytic and neuronal lineages. (B) Pie charts showing the relative proportions of NSCs (blue), NPCs (red), and APCs (green) at indicated time points after MCAO/R. (C) Heatmap displaying the different expression genes between APCs and NPCs. Red indicates upregulated genes and blue indicates downregulated genes. The color intensity is proportional to the normalized expression level of genes. (D) Illustration of *Nrsn1* expression dynamics along the pseudotime trajectory. (E) Heatmap displaying the correlation between different cell types and *Nrsn1*. (F) IF of DCX and Nrsn1 in the striatum at different groups, and arrow pointing to double positive cells in male (left) and female (right) mice. Scale bar: 50 µm. The relative mRNA expression of *Nrsn1* (G) and *Tubb3* (H) post‐OGD/R were measured by RT‐qPCR in C17.2 (left) and primary NSCs (right), n = 4. Data are presented as the mean value ± SEM. **P  < 0.01, ***P  <  0.001.

After confirming the migration of NSCs into the striatum, we focused on their differentiation potential. Quiescent NSCs (qNSCs) and activated NSCs (aNSCs) were identified in the NSCs population [30, 31, 32] (Figure S2A,B). With the increasing reperfusion time, the proportion of qNSCs gradually decreased, while that of aNSCs progressively increased. aNSCs showed significant enrichment of pathways including oxidative phosphorylation and reactive oxygen species signaling, which is indicative of NSC activation and subsequent differentiation (Figure S2C). Pseudotime trajectory analysis likewise placed qNSCs at the starting point, with progressive differentiation into two subsets of aNSCs, verifying that striatum‑migrating NSCs underwent differentiation. NSCs have the capacity to differentiate into astrocytes, neurons, and other cell lineages, with multiple genes and signaling pathways regulating NSC fate decision determination (Figure S2D) [33]. NSCs sequentially differentiate into NPCs and APCs; Figure 2A), and further progressively mature from APCs into mature astrocytes (Figure S2E). Changes in cell number over time further support the differentiation program of NSCs. In the Sham and 0 h groups, NSCs dominated the population, with minimal number of NPCs and APCs. A pronounced transition occurred at 7 days post‐stroke, marked by a robust reduction in NSCs and the reciprocal expansion of both NPCs and APCs, indicating active differentiation of NSCs into these downstream lineages (Figure 2B). Given that post‐stroke neurorepair is largely focused on neuronal recovery and replenishment, we next identified the regulatory genes governing the commitment of NSCs toward NPCs rather than APCs by comparing differentially expressed genes between APCs and NPCs across multiple post‐stroke time points using snRNA‐seq. The analysis revealed that Nrsn1 expression was significantly upregulated in NPCs (Figure 2C; Figure S2F). Notably, Nrsn1‐positive cells were mainly distributed along the differentiation trajectory from NSCs to NPCs (Figure 2D), and Nrsn1 was enriched in the corresponding differentiation pathway (Figure S2F). Previous studies have indicated that Nrsn1 may be involved in NSC differentiation and neuronal maturation [34, 35, 36]. Correlation analysis across cell populations further revealed that Nrsn1 expression was most strongly associated with NPCs (Figure 2E). Analysis of DCX and Nrsn1 expression in the striatum showed that Nrsn1 was expressed in DCX^+^ cells at 7 days post‐stroke in both male and female mice (Figure 2F), consistent with its enrichment in NPCs revealed by pseudotime analysis. These findings further support an association between Nrsn1 expression and the transition of NSCs toward the neuronal progenitor state.

To further investigate the relationship between Nrsn1 expression and NSC differentiation, the murine NSC line NE‐4C and primary mouse NSCs were subjected to oxygen‐glucose deprivation/reoxygenation (OGD/R) to model ischemic injury in vitro [37, 38]. Expression of *Tubb3*, which encodes the neuronal marker Tuj1, was significantly increased following OGD/R (Figure 2G, H), confirming that OGD/R promotes neuronal differentiation of NSCs. *Nrsn1* expression also increased progressively during reoxygenation and became significantly elevated 24 h after reoxygenation (Figure S2G). Taken together, these results indicate that Nrsn1 expression is upregulated during neuronal differentiation of NSCs, supporting a potential role for Nrsn1 in promoting commitment toward the neuronal lineage.

### 2.3 Nrsn1 Overexpression Promotes the Neuronal Differentiation of NSCs in Vitro

**FIGURE 3:**
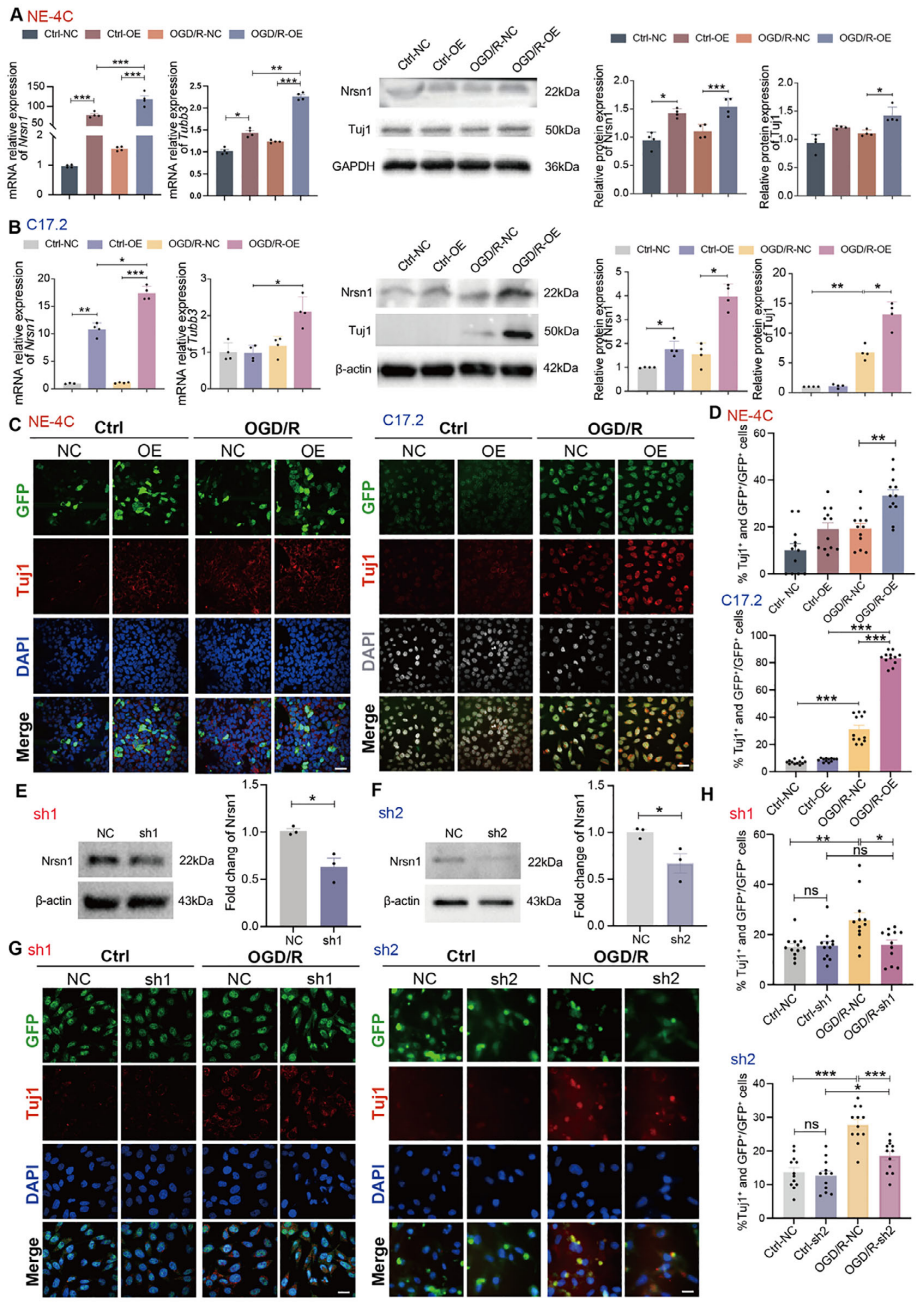
Nrsn1 regulates neuronal differentiation of NSCs in vitro. (A) RT‐qPCR analysis (left) of relative mRNA expression of *Nrsn1* and *Tubb3* in different groups of NE‐4C cells post‐OGD/R. WB (middle) and densitometric quantification (right) of results show the expression of Nrsn1, Tuj1 and GAPDH of NE‐4C cells post‐OGD/R, *n* = 4. NC was generated by inserting a 350‐bp scrambled non‑coding, non‐targeting DNA sequence with the same length as the Nrsn1 coding fragment. (B) RT‐qPCR analysis (left) of relative mRNA expression of *Nrsn1* and *Tubb3* in different groups of C17.2 cells post‐OGD/R. WB (middle) and densitometric quantification (right) of results show the expression of Nrsn1, Tuj1 and β‐actin of C17.2 cells post‐OGD/R, *n* = 4. (C) IF of GFP and Tuj1 in different groups of NE‐4C (left) and C17.2 (right), GFP served as the fusion tag to label OE‐Nrsn1‐NE‐4C or NC cells. (D) Percentage of Tuj1^+^ and GFP^+^ cells among total GFP^+^ cells of NE‐4C (upper) and C17.2 (lower), *n* =  12 images per group. (E) WB and densitometric quantification of results show the expression of Nrsn1 and β‐actin in different groups of sh1‐Nrsn1‐C17.2 cells, *n* = 4. NC consists of a scrambled non‐targeting sequence. (F) WB and densitometric quantification of results show the expression of Nrsn1 and β‐actin in different groups of sh2‐Nrsn1‐C17.2 cells, *n* = 4. (G) IF of GFP and Tuj1 in different groups of sh1‐Nrsn1‐C17.2 (left) and sh2‐Nrsn1‐C17.2 (right), GFP served as the fusion tag to label sh1‐Nrsn1‐C17.2, sh2‐Nrsn1‐C17.2 or NC cells. (H) Percentage of Tuj1^+^ and GFP^+^ cells among total GFP^+^ cells of sh1‐Nrsn1‐C17.2 (upper) and sh2‐Nrsn1‐C17.2 (lower), *n* =   12 images per group. Data are presented as the mean value ± SEM. * *p* < 0.05, **P   <  0.01, ***P  <  0.001, and ns means not significant. Scale bar: 20 µm (C and G).

To investigate whether Nrsn1 regulates the differentiation of NSCs, we first established Nrsn1‐overexpressing (OE‐Nrsn1) stable cell lines in two NSC cell lines, C17.2 and NE‐4C. Nrsn1 was robustly upregulated at both mRNA and protein levels (Figure S3A,B,C), indicating the successful construction of stable overexpression cells. Given that developing neurons exhibit stage‐specific marker expression during maturation [38], we next assessed lineage commitment markers: the NSC marker Nestin (*Nes*) [39] was markedly downregulated, whereas the NPC marker *Pax6* [21, 40], was upregulated in OE‐Nrsn1 cells, confirming the differentiation of NSCs toward neurons (Figure S3D). Relative to untreated controls, OGD/R treatment alone significantly increased both *Nrsn1* and *Tubb3* mRNA and protein levels, relative to the Ctrl groups (Figure 3A,B). Moreover, OE‐Nrsn1 cells exhibited a notably higher expression of Tuj1 at both transcriptional and translational levels than NC cells.

We then identified Tuj1^+^ cells among NE‐4C and C17.2 cells, and the quantitative analysis was conducted by calculating the percentage of and performed quantitative analysis by determining the percentage of Tuj1^+^/GFP^+^ double‑positive cells relative to total GFP^+^ (fusion tag) cells under OGD/R conditions (Figure 3C). Statistical analysis confirmed that OGD/R‐treated OE‐Nrsn1 cells exhibited a significantly higher proportion of Tuj1^+^ cells than in NC group (Figure 3D). Collectively, these findings indicate that Nrsn1 overexpression enhances OGD/R‐induced neuronal differentiation of NSCs.

### 2.4 Nrsn1 Knockdown Attenuates the Neuronal Differentiation of NSCs

To further investigate the role of Nrsn1 in the neuronal differentiation of NSCs, two independent shRNAs (sh1‐Nrsn1 and sh2‐Nrsn1) targeting Nrsn1 were utilized to generate stable knockdown C17.2 and NE‐4C cell lines. Efficient Nrsn1 knockdown at both mRNA and protein levels was validated for both sh1 and sh2 constructs (Figure 3E,F; Figure S3E,F), indicating the successful construction of stable knockdown cell lines. After OGD/R treatment of NC and sh‐Nrsn1, the proportion of Tuj1^+^ cells among GFP^+^ cells were significantly reduced in OGD/R‐treated sh‐Nrsn1‐C17.2 (Figure 3G,H) and sh‐Nrsn1‐NE‐4C cells (Figure S3G,H). These results indicate that neuronal differentiation of NSCs is reduced following Nrsn1 knockdown. Collectively, these data reveal the important role of Nrsn1 in regulating the differentiation of NSCs in vitro.

### 2.5 Foxa2 Transcriptionally Regulates Nrsn1 During Neuronal Differentiation

**FIGURE 4:**
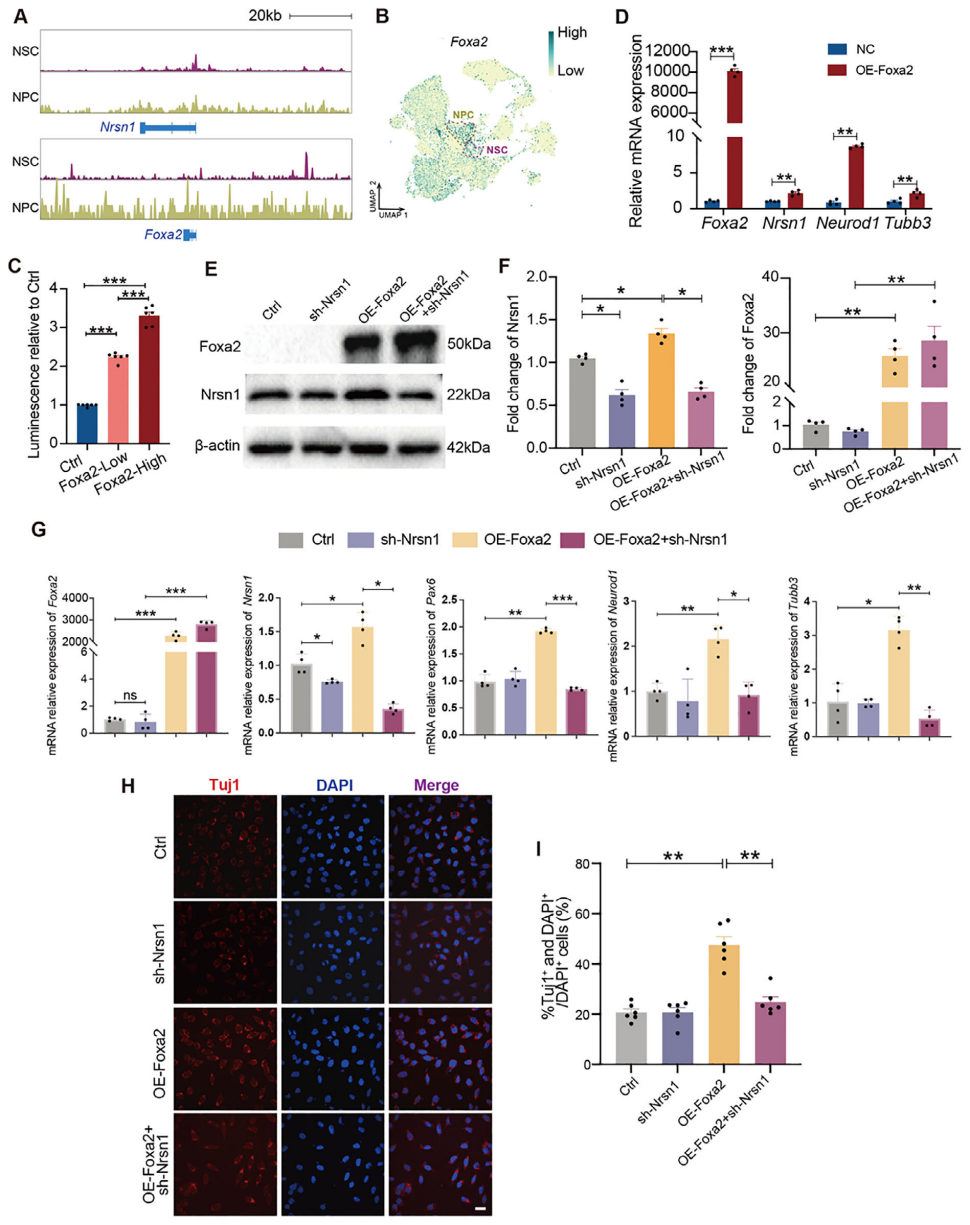
Nrsn1 transcriptionally regulated by Foxa2 mediates the neuronal differentiation of NSCs after OGD/R. (A) Pseudo‐bulk chromatin accessibility profiles for *Nrsn1* and *Foxa2*. Chromosome positions are shown below, with genes on the minus strand in blue. Chromosome coordinates are: Nrsn1 (chr13: 25436022–25453979), and Foxa2 (chr2: 147884797–147888889). (B) snATAC‐seq UMAP visualization of *Foxa2* chromatin accessibility in the NSCs and NPCs. (C) Dual‐luciferase reporter assays demonstrated that Foxa2 overexpression increased Nrsn1 promoter activity. 293T cells were co‐transfected for 48 h with Luc‐Nrsn1 (pGL3‐basic vector containing the ‐2000 to +1 promoter region of Nrsn1), Renilla luciferase (internal control), and pcDNA3‐Foxa2 (Foxa2 expression plasmid), *n* = 6. (D) RT‐qPCR analysis of relative mRNA expression of *Foxa2*, *Nrsn1*, *Neurod1* and *Tubb3* in different groups, *n* = 4. (E) WB and densitometric quantification of results (F) show the expression of Foxa2, Nrsn1 and β‐actin in different groups, *n* = 4. (G) RT‐qPCR analysis of relative mRNA expression of *Foxa2*, *Nrsn1*, *Pax6*, *Neurod1* and *Tubb3* in different groups, *n* = 4. (H) IF of Tuj1 in different groups. (I) Percentage of Tuj1^+^ cells among total cells, *n* = 6 images per group. Data are presented as the mean value ± SEM. * *P*   <  0.05, ** *P*  <  0.01, *** *P*   <  0.001, and ns means not significant. Scale bar: 10 µm.

NSCs require transcriptional reprogramming to re‐enter the cell cycle [41]. To further dissect the molecular regulatory mechanism underlying Nrsn1‐mediated NSC neuronal differentiation, we first identified the upstream transcription factors that target *Nrsn1* using snATAC‐seq. The data revealed increased chromatin accessibility at an upstream region adjacent to Nrsn1 in NSCs and NPCs (Figure 4A), and we further predicted transcription factor binding sites within the Nrsn1 promoter region using JASPAR [42]. The results indicated that multiple Foxa2 binding sites are present in the promoter region of the *Nrsn1* gene (Figure S4A). snATAC‐seq analysis also revealed enhanced chromatin accessibility at a genomic region upstream of Foxa2 in NSCs and NPCs (Figure 4A). To further examine whether Foxa2 directly binds to the endogenous Nrsn1 promoter, we performed ChIP‐qPCR using an anti‐Foxa2 antibody, with IgG as a negative control. Three putative Foxa2‐binding regions within the Nrsn1 promoter were selected based on JASPAR motif prediction. As shown in Figure S4B, significant enrichment of Foxa2‐bound chromatin was detected at Region 2, whereas no significant enrichment was observed at Region 1 or Region 3. These results provide direct evidence that Foxa2 binds to the endogenous Nrsn1 promoter at the predicted Region 2 binding site. Notably, prior studies have shown that Foxa2 is directly regulated in NSCs and functions to induce neuronal differentiation [43], further supporting its potential role in NSC lineage specification. *Foxa2* was highly expressed in NSCs and NPCs. In contrast, *Foxa2* expression decreased sharply in cells undergoing differentiation (Figure 4B). These results demonstrate the stage‐specific expression of *Foxa2*, providing a critical basis for investigating its role in regulating target genes such as *Nrsn1*.

The previous studies have established that *Foxa2* expression modulates stem cell differentiation [44, 45, 46], and its expression is indeed elevated in NSCs during OGD/R‐induced neuronal differentiation (Figure S4C). Therefore, we sought to investigate the regulatory interplay between Foxa2 and Nrsn1 in the context post‐OGD/R. Dual‐luciferase reporter assays demonstrated that Foxa2 overexpression significantly enhanced Nrsn1 promoter activity (Figure 4C). Consistent with these findings, *Foxa2* overexpression not only increased *Nrsn1* mRNA (Figure 4D) and protein levels (Figure 4E,F) in C17.2 cells but also promoted the differentiation of C17.2 cells into neurons, as evidenced by the elevated expression of the neuronal markers *Neurod1* and *Tubb3* (Figure 4D). To determine whether Foxa2 acts as the upstream transcription factor of Nrsn1 and regulates NSC differentiation post‐OGD/R, we generated four C17.2 cell lines: Ctrl, sh‐Nrsn1, OE‐Foxa2, and sh‐Nrsn1+OE‐Foxa2. We verified the efficiency of Foxa2 overexpression and Nrsn1 knockdown in respective groups (Figure 4E,F). Critically, Foxa2 overexpression robustly upregulated Nrsn1 expression, whereas Nrsn1 knockdown had no effect on Foxa2 protein levels, validating Foxa2 as the bona fide upstream regulator of Nrsn1.

We next examined the key neuronal differentiation markers (Figure 4G), the results showed that OE‐Foxa2 significantly upregulated the neural progenitor marker *Pax6*, the early neuronal marker *Neurod1*, and immature neuronal marker *Tubb3*. These effects were reversed by Nrsn1 knockdown: *Pax6*, *Neurod1* and *Tubb3* were reduced in sh‐Nrsn1+OE‐Foxa2 group relative to OE‐Foxa2 group. Furthermore, OE‐Foxa2 significantly increased the proportion of Tuj1^+^ cells (Figure 4H,I). This pro‐differentiation effect was abrogated by Nrsn1 knockdown, which was comparable to Ctrl group. Collectively, these data demonstrate that Foxa2 promotes NSCs neuronal differentiation under OGD/R conditions with Nrsn1‐dependent manner.

### 2.6 Nrsn1 Interacts With Smarcc1 and Facilitates Its Nuclear Translocation to Promote NSCs Differentiation

**FIGURE 5:**
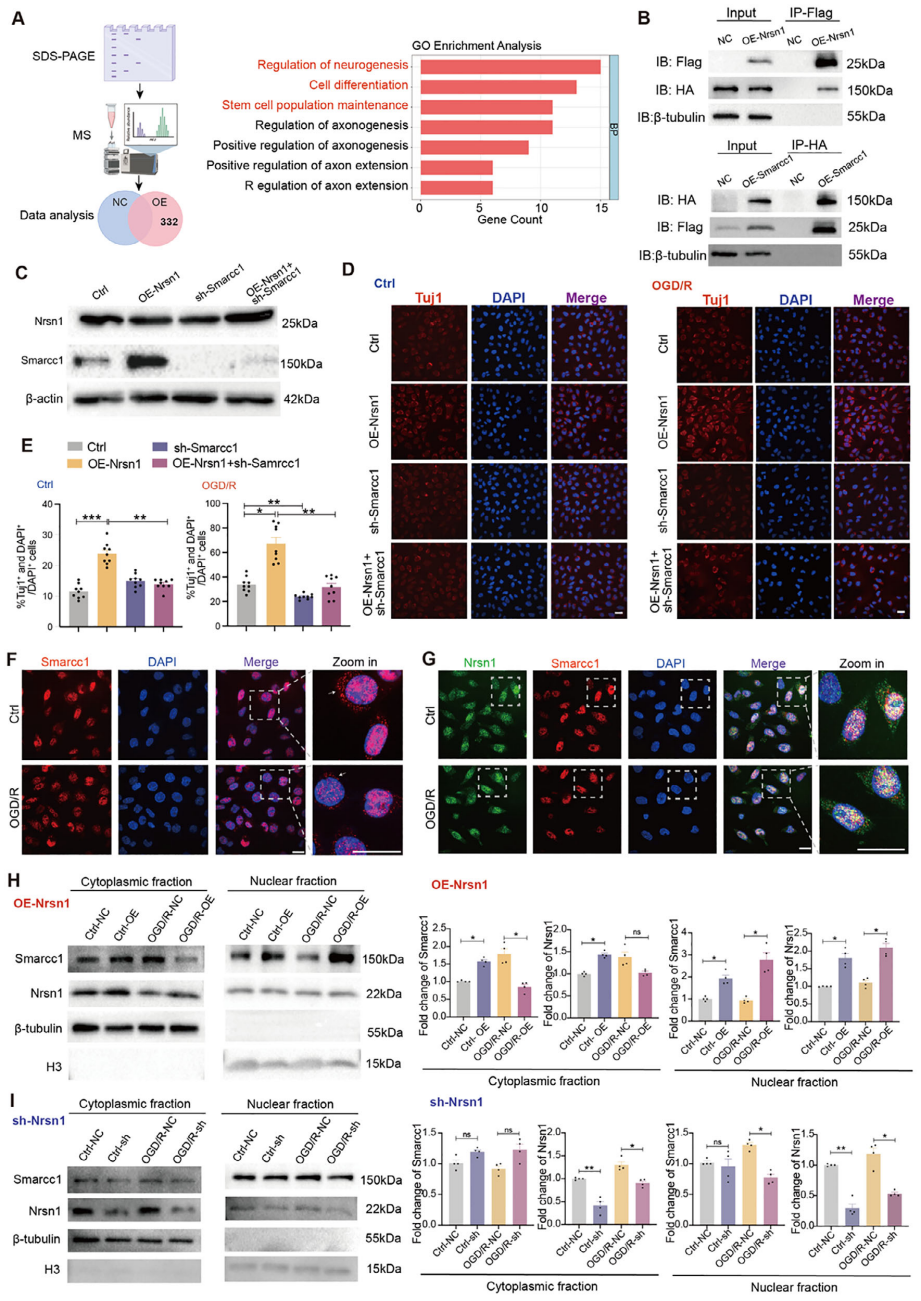
Nrsn1 interacts with Smarcc1 and facilitates its nuclear translocation to promote NSCs differentiation after OGD/R. (A) Schematic workflow of the IP‐MS assay (left). The LC–MS interactome analysis was performed using C17.2 cells overexpressing Flag‐tagged Nrsn1, with corresponding NC cells used as the negative control. GO analysis of differentially expressed proteins in the OE group with respect to biological processes (BP; right). (B) co‐IP assays were performed in NSCs transfected with Flag‐tagged Nrsn1 overexpression plasmid (OE‐Nrsn1) or NC (upper). co‐IP assays were performed in NSCs transfected with HA‐tagged Smarcc1 overexpression plasmid (OE‐Smarcc1) or NC (lower). (C) WB results show the expression of Smarcc1, Nrsn1 and β‐actin. (D) IF of Tuj1 in different groups under Ctrl (left) and OGD/R (right) conditions. Scale bar: 10 µm. (E) Percentage of Tuj1^+^ cells among total cells under Ctrl (left) and OGD/R (right) conditions, *n* = 9 images per group. (F) IF of Smarcc1 in C17.2 cells under Ctrl and OGD/R conditions. The arrow indicates Smarcc1 in the cytoplasm. (G) IF of Nrsn1 (GFP/Green) and Smarcc1 (Red) in C17.2 cells under Ctrl and OGD/R conditions. Scale bar: 20 µm. (H) WB and densitometric quantification show the expression of Smarcc1, Nrsn1, β‑tubulin and H3 in cytoplasmic and nuclear fractions in OE‐Nrsn1‐C17.2. Protein levels were normalized to β‑tubulin for the cytoplasmic fraction and H3 for the nuclear fraction, *n* = 4. (I) WB and densitometric quantification show the expression of Smarcc1, Nrsn1, β‑tubulin and H3 in cytoplasmic and nuclear fractions in sh‐Nrsn1‐C17.2. Protein levels were normalized to β‑tubulin for the cytoplasmic fraction and H3 for the nuclear fraction, *n* = 4. Data are presented as the mean value ± SEM. * *P*  <  0.05, ** *P*  <  0.01, *** *P*  <  0.001, and ns means not significant.

To identify the interactors of Nrsn1 in NSCs, we performed immunoprecipitation coupled with mass spectrometry (IP‐MS) and identified 332 Nrsn1‐binding proteins (Figure 5A; Figure S5A,B; Table S5). These proteins were identified based on exclusive detection in the OE‐Nrsn1 pulldown compared with the NC control, Global.PG.Q.Value ≤0.01, and at least one unique peptide. As an additional sensitivity analysis, we applied a more stringent requirement of at least two unique peptides, which reduced the number of candidate proteins from 332 to 68 (Table S6). Gene Ontology (GO) functional enrichment analysis of the 332 proteins revealed enrichment of biological processes related to “cell differentiation” and “stem cell population maintenance” (Figure 5A; Figure S5C) [47]. Importantly, GO analysis of the 68 proteins retained after the stringent peptide‐level filtering showed broadly consistent functional enrichment patterns, including tissue regeneration and microtubule‐related processes (Figure S5D). Smarcc1 (BAF155) was identified under both the initial and more stringent filtering criteria, supported by four unique peptides, 3.62% sequence coverage, and a Global.PG.Q.Value of 0.000336, and was exclusively detected in the OE‐Nrsn1 pulldown. Notably, Smarcc1‐associated functions were also represented among the GO‐enriched terms in the stringent 68‐protein dataset, further supporting the biological relevance of Smarcc1 as a candidate Nrsn1‐associated protein. These observations led us to hypothesize that Smarcc1 may mediate the pro‐differentiation effects of Nrsn1 in NSCs. Smarcc1 is a core subunit of the BAF complex, which has been shown to remodel chromatin structure and regulate the transcription of lineage‐specific genes to control stem cell differentiation across multiple systems [48, 49, 50]. Reciprocal co‐IP assays further confirmed the specific interaction between Nrsn1 and Smarcc1 (Figure 5B). Next, we established Smarcc1‐OE‐C17.2 and Nrsn1‐OE‐C17.2 cells, with overexpression efficiency confirmed (Figure S5E–H). Intriguingly, Nrsn1 overexpression had no impact on Smarcc1 mRNA levels (Figure S5F), but significantly upregulated Smarcc1 protein expression (Figure 5C; Figure S5G). In contrast, Smarcc1 overexpression did not alter Nrsn1 expression (Figure S5H). Then we evaluated neuronal differentiation by quantifying Tuj1^+^ cells in OE‐Nrsn1 and OE‐Nrsn1+sh‐Smarcc1 cells after OGD/R (Figure 5D,E). OE‐Nrsn1 significantly increased the proportion of Tuj1^+^ cells; however, this pro‐differentiation effect was abrogated in OE‐Nrsn1+sh‐Smarcc1 cells. These results further indicate that Nrsn1 interacts with Smarcc1.

Given that the BAF complex functions in the nucleus [51, 52], we next examined the subcellular localization of Smarcc1 following OGD/R treatment. OGD/R promoted the nuclear accumulation of Smarcc1 in both C17.2 cells (Figure 5F) and primary NSCs (Figure S5I). Nrsn1 overexpression further enhanced Smarcc1 nuclear translocation in C17.2 cells (Figure 5G,H), implying that Nrsn1 may facilitate the nuclear transport of Smarcc1 to drive neuronal differentiation of NSCs. Conversely, Nrsn1 knockdown led to increased cytoplasmic expression and decreased nuclear expression of Smarcc1, which confirmed our speculation (Figure 5I). These findings indicate that Nrsn1 promotes neuronal differentiation by facilitating Smarcc1 nuclear translocation.

### 2.7 Nrsn1 Promotes Neurogenesis Post‐Stroke to Enhance Neurological Recovery

**FIGURE 6:**
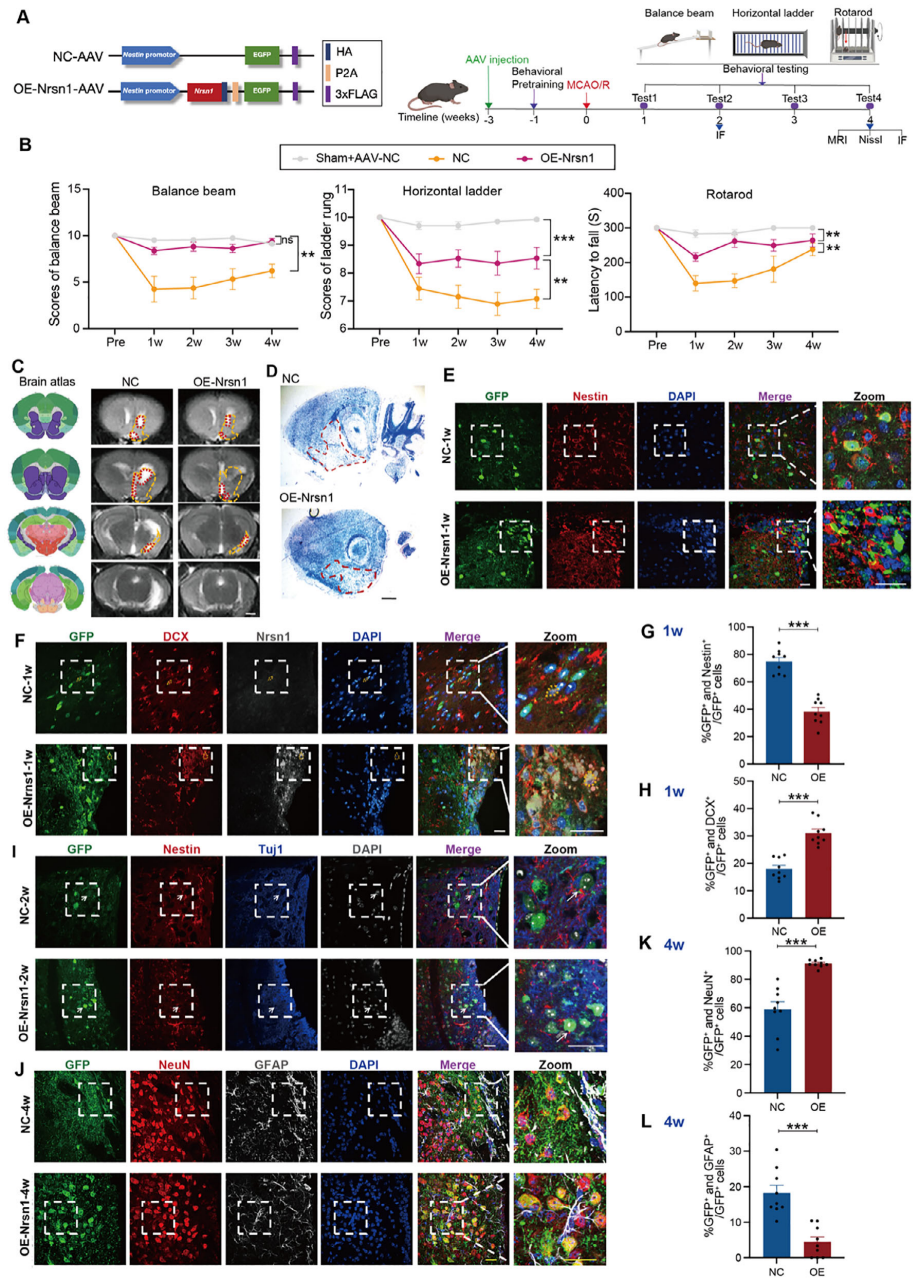
Nrsn1 overexpression promotes NSCs differentiation into neurons in the brains post‐stroke. (A) Schematic diagram of the rAAV vector construct used in this study (left). Summary of the experimental timeframes (right). (B) Behavioral test results (the balance beam, ladder rung and rotarod tests) at pre, 1 to 4 weeks after MCAO/R, *n* = 10. The mice in the Sham + AAV‐NC control group were only subjected to intracerebral injection of control AAV‐NC virus with Sham surgery. (C) MRI images show brain cerebral infarct at 4 w after MCAO/R. The brain atlas illustrates the anatomical localization of the striatum, and the striatum is marked by yellow dotted lines in MRI image. The infarct area is marked by red dotted lines. Scale bar: 2 mm. (D) Nissl staining of the brain at 4 w post‐stroke, and the infarct area is marked by dotted lines. Scale bar: 2 mm. (E) IF of GFP and Nestin in different groups at 1 w post‐stroke. (F) IF of GFP, DCX and Nrsn1 in different groups at 1 w post‐stroke. (G) Percentage of GFP^+^ and Nestin^+^ NSCs among total GFP^+^ NSCs of 1 w after stroke. (H) Percentage of GFP^+^ and DCX^+^ NSCs among total GFP^+^ NSCs of 1 w post‐stroke. (I) IF of GFP, Nestin and Tuj1 in different groups at 2 w post‐stroke. (J) IF of GFP, NeuN and GFAP in different groups at 4 w post‐stroke. (K) Percentage of GFP^+^ and NeuN^+^ NSCs among total GFP^+^ NSCs at 4 w post‐stroke. (L) Percentage of GFP^+^ and GFAP^+^ NSCs among total GFP^+^ NSCs at 4 w post‐stroke, *n* = 9 images per group. Data are presented as the mean value ± SEM. * *P*   < 0.05, ** *P*  < 0.01, *** *P*  < 0.001, and ns means not significant. Scale bar: 50 µm.

To evaluate the potential role of Nrsn1 in the regulation of neurogenesis post‐stroke, mice were subjected to Sham or MCAO/R with or without Nrsn1 overexpression in the endogenous NSCs (Figure 6A). Nrsn1 was specifically overexpressed in adult NSCs via an Adeno‐associated virus serotype (AAV)2/9 vector driven by the *Nestin* promoter [38], and we verified that AAV injection delivered GFP and Nrsn1 to the NSCs and that there was a good overlap between GFP and Nestin [53] (Figure S6A). Neurological functions were evaluated by balance beam, ladder rung and rotarod test after MCAO/R (Figure 6B). Beam walking tests revealed that, starting on the 1th week post‐stroke, the OE‐Nrsn1 group exhibited significantly fewer falls and gradually increased test scores. By 4 w post‐stroke, motor function in the OE‐Nrsn1 group was nearly restored to Sham + AAV‐NC group. Concordant results were obtained via ladder rung tests and rotarod test. The OE‐Nrsn1 group showed marked recovery of rotarod performance by 2 w post‐stroke, which persisted until 4 w. These findings indicate that overexpression of Nrsn1 promotes better functional recovery after stroke.

At 4 weeks post‐stroke, Magnetic resonance imaging (MRI) showed that OE‐Nrsn1 mice exhibited significantly smaller infarct areas compared with NC mice (Figure 6C). Nissl staining further confirmed that infarct volume was markedly reduced in the OE‐Nrsn1 group, consistent with the improved neurological recovery (Figure 6D). These functional and morphological findings provide the strong rationale for further exploring the differentiation status of endogenous NSCs in the ischemic brain.

Proliferating neuroblasts generated in the SVZ eventually migrate to the ischemic lesion site to differentiate into newly formed neurons, thereby facilitating post‐stroke neural repair [54]. Our results showed that the number of GFP^+^/Nestin^+^ double‐positive cells was significantly lower in the OE‐Nrsn1 group than in the NC group (Figure 6E,F), whereas the number of GFP^+^/ DCX^+^ double‐positive cells was markedly higher in the OE‐Nrsn1 group on the 1th week post‐stroke (Figure 6G,H). These findings suggest that NSCs in the OE‐Nrsn1 group tend to differentiate more preferentially into NPCs. Then we analyzed the expression of specific markers at 2 and 4 w post‐MCAO/R. At 2 w post‐MCAO/R, the number of GFP^+^/Nestin^+^ double‐positive cells remained significantly lower in the OE‐Nrsn1 group, while GFP^+^/DCX^+^ double‐positive cell counts were also markedly reduced (Figure 6I; Figure S6B,C). Concomitantly, GFP^+^/Tuj1^+^ double‐positive cells were elevated in the OE‐Nrsn1 group, indicating an enhanced tendency of NSCs to differentiate toward the neuronal lineage. At 4 weeks post‐stroke, GFP^+^/Tuj1^+^ double‐positive cell numbers in the OE‐Nrsn1 group remained significantly higher than NC group, whereas the number of GFP^+^/DCX^+^ double‐positive cells was comparable between the two groups, with no significant difference (Figure S6D,E). To further define the terminal differentiation fate, we assessed mature lineage markers and found that the OE‐Nrsn1 group exhibited a significantly higher number of GFP^+^/NeuN^+^ double‐positive cells (mature neurons, Figure 6J,K) but a lower number of GFP^+^/GFAP^+^ double‐positive cells (astrocytes, Figure 6J,L) relative to the NC group. Collectively, these data demonstrate that Nrsn1 overexpression directs the differentiation of NSCs toward neurons rather than astrocytes following ischemic stroke.

### 2.8 Nrsn1‐Overexpressing NSCs Transplantation Promotes Neural Repair in MCAO/R Mice

**FIGURE 7:**
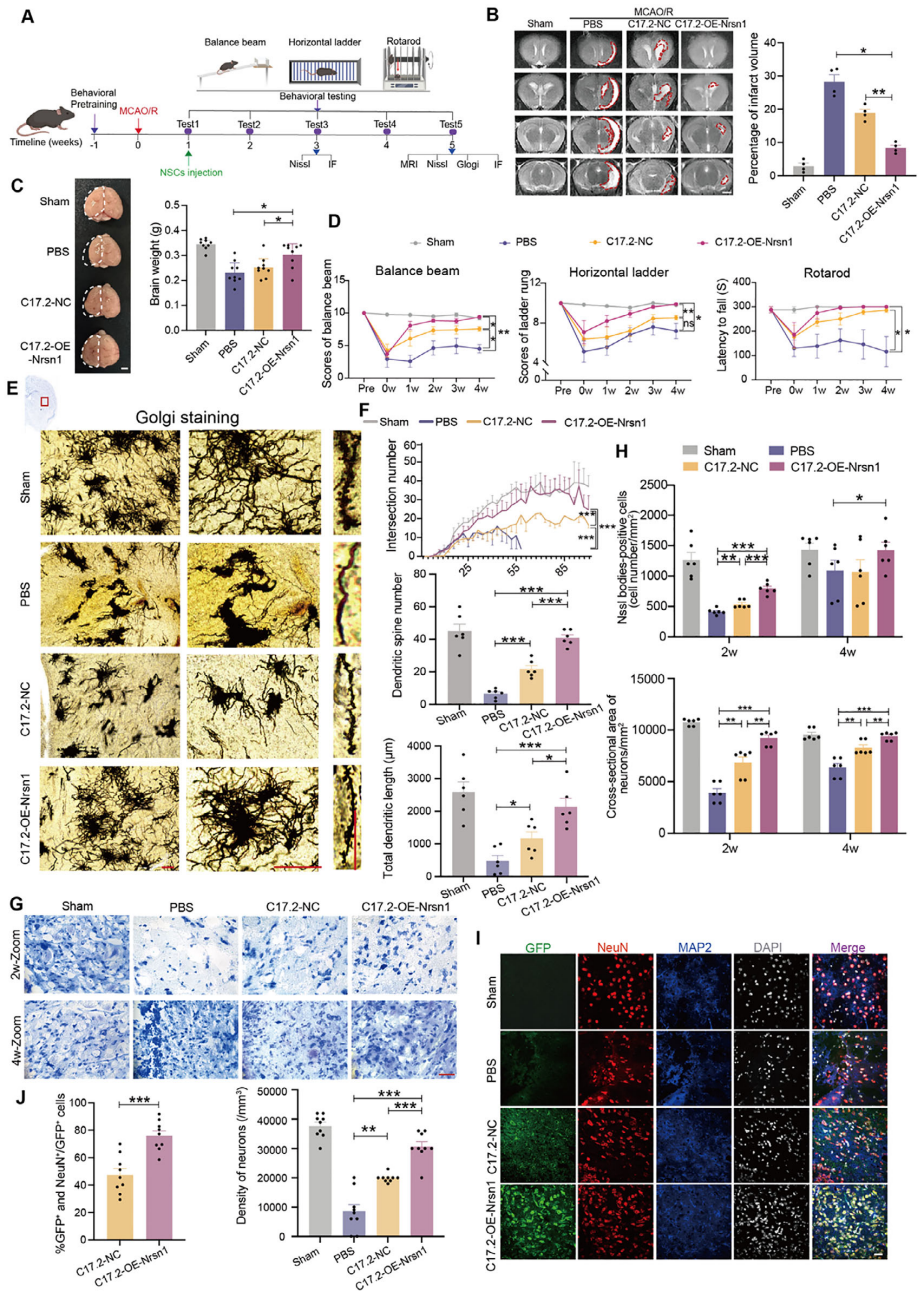
Therapeutic effects of Nrsn1‐Overexpressing NSCs transplantation in MCAO/R. (A) Summary of the experimental timeframes. (B) MRI images and quantification of infarct volume show brain cerebral infarct at 4 w after treatment. The infarct area is marked by dotted lines, *n* = 4. Scale bar: 2 mm. (C) Representative images (left) and quantification (right) of the brain weights show brain weight at 4 w after treatment. The ischemic hemispheres are marked by dotted lines, *n* = 9. Scale bar: 2 mm. (D) Behavioral test results (the balance beam, ladder rung, rotarod tests) at pre, 0 to 4 weeks after treatment, *n* = 10. (E) Representative images of Golgi‐Cox staining in the infarct area at 4 w after treatment. Scale bar: 50 µm. (F) Quantitative analysis of neuronal complexity (upper), dendritic spine number (middle), and total dendritic length (lower), *n* = 6. (G) Nissl staining of infarct area in the brain at 2 and 4 w after treatment. Scale bar: 25 µm. (H) Nissl bodies positive cells number per mm^2^, and cross‐sectional areas of neurons per mm^2^, *n* = 6. (I) IF of GFP, NeuN and MAP2 in different groups at 4 w post‐stroke. Scale bar: 50 µm. (J) Percentage of GFP^+^ and NeuN^+^ NSCs among total GFP^+^ NSCs of 4 w after stroke in mice (left), *n* = 9 images per group. Density of neurons per mm^3^ (right), *n* = 9 images per group. Data are presented as the mean value ± SEM. *P < 0.05, ** *P*< 0.01, *** *P* < 0.001, and ns means not significant.

In vivo cell transplantation is widely regarded as the gold standard in stem cell biology for interrogating the developmental potential of donor cells [55]. Given the stronger neuronal differentiation capacity of OE‐Nrsn1‐C17.2 cells, we transplanted these cells into MCAO/R mice to assess their therapeutic potential (Figure 7A). Compared to the severe damages of brain tissues in C17.2‐NC mice, C17.2‐OE‐Nrsn1‐transplanted mice showed significantly reduced infarct areas by MRI (Figure 7B), which was further confirmed by the results of brain weight analysis (Figure 7C). Starting at 2 weeks post‐treatment, mice transplanted with C17.2‐OE‐Nrsn1 cells exhibited significantly fewer falls and higher performance scores than those receiving control C17.2 cells in the balance beam tests. By 4 weeks post‐treatment, the C17.2‐OE‐Nrsn1 group also showed markedly superior motor performance in the ladder rung tests, indicating that C17.2‐OE‐Nrsn1 transplantation significantly promotes motor function recovery in MCAO/R mice (Figure 7D). In the rotarod test, NSC transplantation prolonged the latency to fall of MCAO/R mice, reflecting progressive motor function recovery. These results indicated C17.2‐OE‐Nrsn1 group achieved substantial motor recovery by 2 weeks post‐treatment, which was stably maintained until 4 weeks, whereas the control group had a slower recovery rate.

At 4 weeks post‐MCAO/R, C17.2‐OE‐Nrsn1 transplantation not only reduced infarct volume but also improved neuronal structural complexity in the striatum, as evidenced by increased dendritic density and length, reduced dendritic spine loss, and enhanced complexity of neuronal projections (Figure 7E,F). Nissl staining showed that neuronal damage and loss were markedly alleviated in the C17.2‐OE‐Nrsn1 group compared with the NC group after MCAO/R (Figure 7G; Figure S7A). Notably, a significant increase in Nissl‐positive cells was observed in the C17.2‐OE‐Nrsn1 group [56] (Figure 7H). These findings indicate that transplantation of Nrsn1‐overexpressing NSCs confers superior therapeutic efficacy in mitigating ischemic stroke‐induced neuronal damage.

Additionally, the C17.2‐OE‐Nrsn1 group displayed more GFP^+^/NeuN^+^ double‐positive cells (mature neurons, Figure 7I,J) and fewer GFP^+^/GFAP^+^ double‐positive cells (astrocytes) compared with the control group (Figure S7B,C). MAP2 expression further confirms that neuronal integrity is stronger in the C17.2‐OE‐Nrsn1 group (Figure 7I). The average optical density of neurons in the striatum was also significantly increased in mice transplanted with C17.2‐OE‐Nrsn1 cells at 4 weeks post‐MCAO/R [38, 56] (Figure 7J). Collectively, these results demonstrate that C17.2‐OE‐Nrsn1 NSC transplantation elicits the more robust neurorestorative effect than unmodified NSCs, highlighting the critical role of Nrsn1 in enhancing post‐stroke neurogenesis and facilitating neurological recovery.

To the best of our knowledge, the present study demonstrated for the first time that Nrsn1 overexpression in NSCs can lead to beneficial phenotypic changes in the brain post‐stroke. Specifically, our data revealed that Foxa2 transcriptionally regulates Nrsn1, and Nrsn1 further facilitates the nuclear translocation of Smarcc1 to promote the neuronal differentiation of NSCs (Figure S8). Collectively, the Foxa2‐Nrsn1‐Smarcc1 axis may represent a promising therapeutic strategy to enhance neuronal differentiation and promote functional recovery following stroke.

## 3 Discussion

Unlike lower vertebrates (e.g., zebrafish [57] and salamanders [29]) that retain robust regenerative power post‐brain injury, mammals struggle to achieve effective recovery due to their inherently limited regenerative capacity [58]. Current clinical treatments for stroke mainly focus on recanalization and neuroprotection, but fail to address the limited endogenous neurogenesis in adult brains [59, 60]. Nrsn1 is highly enriched in the developing chick brain and persists into adulthood, and its localization to motor neuron somata and regenerating axon terminals implies a role in axonal regeneration [34, 36]. While Nrsn1 has been implicated in axonal regeneration, its role in post‐stroke neurogenesis remained unclear. Our study demonstrates that Nrsn1 facilitates neurogenesis and neurological recovery in the chronic phase after stroke, thus providing a promising therapeutic target for brain injury repair and long‐term functional outcomes, which represents an unmet clinical need in stroke treatment.

Brain regeneration requires spatiotemporal coordination of complex biological responses [29]. Integrating snRNA‐seq, snATAC‐seq, and Stereo‐seq, we characterized the post‐stroke mouse brain's transcriptional and epigenomic landscape, with a focus on networks underlying NSC neuronal differentiation. Consistent with prior reports that upregulated ribosomal signatures mark the NSC‐to‐NPC transition [61], our data showed elevated ribosomal and mitochondrial components in NPCs (mitochondrial genes retained during library preparation), validating the reliability of cell type annotation. Existing literature has also reported on astrocyte transdifferentiation dynamics [62, 63]. Reactive astrocytes in the damaged striatum undergo transcriptional reprogramming, shifting toward a neural progenitor‐like state, which may contribute to endogenous repair by supplementing NPC pools. This transdifferentiation process was tightly linked to NSCs differentiation trajectories, suggesting crosstalk between astrocytes and NSCs in the post‐stroke microenvironment. These findings provide the targets and time windows for developing targeted stroke therapies.

Endogenous NSCs are activated post‐stroke, migrate to the striatum, and differentiate into neural lineage cells to mediate ischemic tissue repair [18, 19, 20], though this process is suppressed in the robust inflammatory microenvironment of intracerebral hemorrhage models [64]. Our MCAO/R model with 60 min of ischemia avoided inflammatory storms, thus enabling NSC survival and neurogenesis. Clinically, stroke causes substantial disability and mobility impairment [10, 65], underscoring the urgency to identify pro‐neurogenic regulators. Consistent with our findings, Tlx overexpression enhances post‐stroke neurogenesis and motor recovery even in aged mice [66], while the miR‐449b/Notch1 and miR‐148b/Wnt pathways also regulate NSC fate and functional recovery [67, 68]. Temporal dynamics of striatal NSC migration and differentiation further support our experimental design [69, 70, 71]. Notably, the Wnt and Notch pathways are well‐established regulators of NSCs fate during post‐stroke neurogenesis. Whether Nrsn1 interacts with these pathways deserves further investigation. For instance, by modulating Smarcc1‐mediated chromatin remodeling of Wnt/Notch target genes. Such crosstalk could form a complex regulatory network that fine‐tunes neurogenesis post‐stroke.

Nrsn1 is the only known membrane protein that harbors a microtubule‐binding domain. Like the microtubule‐associated protein Tau [72], Nrsn1 may regulate NSC differentiation by modulating microtubule dynamics. This unique dual identity that combines membrane localization with microtubule binding distinguishes Nrsn1 from other neurogenic regulators, as it enables the integration of membrane signaling cascades with cytoskeletal remodeling, which is a key process for NSC migration and differentiation.

A key focus of our study is the functional interaction between Nrsn1 and Smarcc1. As a SWI/SNF complex component, Smarcc1 promotes stem cell differentiation via chromatin remodeling to repress Nanog expression [73], and its nuclear translocation is regulated by KPNA2 in bladder cancer [74]. Our bioinformatic analyses (using DeepLoc 2.0 and LocNES) revealed that Nrsn1 harbors both a nuclear localization signal and a nuclear export signal (Figure S9). Moreover, Nrsn1 overexpression promoted Smarcc1 nuclear translocation and increased Smarcc1 protein abundance without affecting its mRNA expression levels (Figure 5C; Figure S5F). Nrsn1 is a typical membrane/synapse‐associated protein that localizes to the neuronal plasma membrane and postsynaptic density, playing a fundamental role in neuronal morphogenesis and synaptic signal transmission. In contrast, Smarcc1, as a core subunit of the SWI/SNF chromatin remodeling complex, serves as a key component of the intracellular transcriptional regulatory network and directly participates in the transcriptional programs governing stem cell fate decisions by modulating the chromatin accessibility of target genes. Notably, the present study demonstrated that Nrsn1 physically interact with Smarcc1 and facilitate its nuclear translocation. This molecular event ingeniously bridges neuronal structural signals with stem cell fate regulatory programs, thereby establishing a trans‐spatial regulatory bridge model that links extracellular microenvironmental stimuli or intracellular functional status sensing to nuclear transcriptional decision‐making. This model not only reveals the critical mechanism underlying the role of Nrsn1 in neural stem cell differentiation after stroke but also elucidates a paradigm through which membrane‐associated proteins transmit regulatory signals into the nucleus to guide stem cell fate choices.

We next examined whether the increased Smarcc1 protein level induced by Nrsn1 results from altered protein stability or ubiquitin–proteasome‐dependent degradation. Neither the CHX chase assay nor MG132 treatment revealed differences in Smarcc1 protein turnover between control and Nrsn1‐overexpressing cells (Figure S10), suggesting that Nrsn1 does not regulate Smarcc1 through these mechanisms. Instead, our findings support a model in which Nrsn1 interacts with Smarcc1 in the cytoplasm and facilitates its nuclear translocation. The increase in Smarcc1 protein abundance without a corresponding change in mRNA levels also raises the possibility that additional post‐transcriptional or post‐translational mechanisms contribute to this process, which warrants further investigation.

However, this study had several limitations. First, the mechanisms underlying the dynamic changes of Nrsn1 in the brain post‐stroke remain unresolved. Second, the impact of Nrsn1 on neurological behavior and NPC migration during the chronic phase post‐stroke requires deeper investigation. Specifically, tracking NPC migration trajectories with Nrsn1 manipulation could clarify whether Nrsn1 affects migration efficiency or directional specificity to the infarct core. Third, we exclusively used young adult male mice in all treatment assays to eliminate confounding effects from female sex hormones and the estrous cycle, which have been documented to markedly alter ischemic brain injury outcomes [75, 76]. However, stroke disproportionately occurs in elderly humans, whose brains possess unique regenerative, inflammatory, and vascular signatures [33, 77, 78] that could modify the expression and function of Nrsn1. Accordingly, follow‐up research utilizing aged and female mouse models is essential to improve the translational relevance of our present findings.

## 4 Conclusion

Here, by integrating single‐cell multi‐omics and spatial transcriptomics, we systematically delineated cell type‐specific spatiotemporal alterations in the striatum following ischemic stroke. We identified Nrsn1 as a markedly upregulated gene during neuronal differentiation of NSCs in the post‐ischemic recovery phase. We further showed that modulating Nrsn1 expression directly dictates the neuronal differentiation ratio of NSCs. Mechanistically, Foxa2 directly promotes Nrsn1 transcription, and Nrsn1 facilitates neuronal differentiation by enhancing the nuclear translocation of Smarcc1. Finally, both endogenous neurogenesis and transplantation of Nrsn1‐overexpressing NSCs significantly promoted neural repair and functional recovery in MCAO/R mice. Our findings establish Nrsn1 as a critical regulator of neuronal lineage commitment in NSCs and offer a novel target for manipulating NSC fate to improve stroke therapy.

## 5 Experimental Section

### 5.1 Animal Experiment

Male and female C57BL/6 mice (age: 7−8 weeks, weight: 21−24 g) were employed for this study. All animal procedures were performed in compliance with guidelines for the care and use of animals and were approved by the Huazhong Agriculture University Institutional Animal Care and Use Committee (approval number: HZAUMO−2024−0024). Mice were assigned into groups subjected to MCAO/R with various recovery periods (0 h, 24 h, 7 d and 14 d) or Sham operation.

### 5.2 MCAO/R Model Establishment

Ischemic stroke was established with MCAO/R surgery on C57BL/6 mice according to the laboratory's preliminary experimental methods [1]. Briefly, the mice were anesthetized with 2% isoflurane (RWD, China), and were placed in the supine position. To induce focal cerebral ischemia, a silicon‐coated filament (RWD, China) was inserted into the left middle cerebral artery (MCA) to block blood flow. After 60 min later, the filament was extracted for reperfusion. Rectal temperature was maintained at 37°C during the entire procedure. Mice with the body weight of less than 14 g within 7 days after surgery were excluded from the study. Mice of the Sham‐operated group were performed the same as the MCAO/R procedure without filament insertion. All mice were randomly allocated into different groups following a strict randomization protocol. The randomization process was conducted by an independent researcher who was not involved in the subsequent experimental operation and data collection to avoid selection bias.

### 5.3 Brain Tissue Dissection

Following MCAO/R surgery, mice were allowed to recover for 0 h, 24 h, 7 d, or 14 d. Both MCAO/R and sham‐operated mice were deeply anesthetized with 2% isoflurane and euthanized by cervical dislocation. Brains were rapidly removed. For snRNA‐seq and snATAC‐seq, the ipsilateral striatum was carefully dissected using ophthalmic forceps. All samples were processed on ice, snap‐frozen in liquid nitrogen, and stored at −80°C until use. For Stereo‐seq, brains were embedded in optimal cutting temperature (OCT) compound and stored at −80°C.

### 5.4 Immunofluorescence Staining

For IF of mouse brain tissues, mice were anesthetized and immediately perfused with PBS, followed by 4% Paraformaldehyde for 30 min. Brains were fixed overnight in fixative at 4°C. Fixed brains were dehydrated in 30% sucrose in PBS for 2 days at 4°C. Brains were embedded in the OCT compound. Coronal or sagittal brain sections (25 µm thick) were obtained using a cryostat microtome. Tissues were permeabilized, blocked, and incubated as the above‐mentioned protocol for cultured cell staining. Tissues were incubated with primary antibodies, including anti‐Nestin (1:50, Santa, sc‐23927); anti‐Tuj1 (1:100, abcam, ab18207); anti‐DCX (1:200, abcam, ab135349); anti‐Nrsn1 (1:100, Invitrogen, PA5‐98607); GFP (1:2000, abcam, ab13970); NeuN (1:100, abcam, ab177487); anti‐GFAP (1:1000, abcam, ab4648); anti‐MAP2 (1:200, Proteintech, 67015‐1‐Ig) and anti‐Smarcc1 (1:100, abcam, ab305037). The cells were planted on round glass coverslips, and fixed with 4% paraformaldehyde overnight at 4°C, then permeabilized with 0.25% Triton X‐100, and blocked with 2% BSA for 1 h at room temperature. The coverslips were incubated with primary antibodies at 4°C overnight. The primary antibodies were then washed off and sections were incubated with secondary antibodies for 1 h at room temperature. Cells were counterstained with DAPI for 10 min after washing. Images were captured using a spinning disk confocal microscope (Andor Technology, UK).

### 5.5 Nuclei Isolation for snRNA‐seq and snATAC‐seq

Nuclei were isolated from frozen striatal tissues using a mechanical dissociation protocol as previously described. Briefly, frozen samples were thawed on ice and homogenized in pre‐chilled homogenization buffer using Dounce homogenizers. Homogenates were filtered through 40‐µm cell strainers and centrifuged at 1,200 × g for 5 min at 4°C. Nuclear pellets were resuspended in blocking buffer containing 1% BSA and 0.2 U/µL RNase inhibitor, followed by an additional centrifugation at 500 × g for 5 min. Purified nuclei were resuspended in 1% BSA for downstream library construction.

### 5.6 snRNA‐seq Library Construction and Sequencing

Single‐nucleus RNA‐seq libraries were prepared using the DNBelab C Series High‐throughput Single‐Cell Library Preparation Kit (BGI, China) according to the manufacturer's protocol. Single‐nucleus suspensions were loaded onto microfluidic chips for droplet generation and incubated at room temperature for 20 min to allow mRNA capture. After emulsion breaking and bead recovery, reverse transcription and cDNA amplification were performed. DNA nanoballs were generated from purified PCR products and sequenced on the MGI DNBSEQ‐Tx platform with read lengths of 41 bp (Read 1), 100 bp (Read 2), and 10 bp (index).

### 5.7 snATAC‐Seq Library Preparation and Sequencing

For snATAC‐seq library preparation, the DNBelab C Series Single‐Cell ATAC Library Prep Kit (MGI, China) was used. Library construction was performed according to the manufacturer's instructions, including key steps: transposition, droplet formation, PCR pre‐amplification, emulsion disruption, capture bead collection, DNA amplification, and final purification. Purified libraries were sequenced on the MGI DNBSEQ‐T1 platform with the following parameters: Read 1 (109 bp), Read 2 (50 bp), and sample index (10 bp). The library quality was strictly controlled before sequencing. Library concentration was quantified using the Qubit ssDNA Assay Kit to ensure DNA molecule numbers and concentrations met sequencing requirements. Additionally, library fragment size distribution was analyzed using the Agilent 2100 Bioanalyzer system (Agilent Technologies, China).

### 5.8 Stereo‐seq Library Preparation and Sequencing

Stereo‐seq libraries were constructed using the STOmics Gene Expression Kit S1 (BGI, China) according to the manufacturer's protocol (version 1.1) with minor modifications. Coronal brain sections (15 µm thick) were mounted onto Stereo‐seq chips and fixed in methanol at −20°C for 30 min. After nucleic acid staining and imaging, tissue sections were permeabilized at 37°C for 12 min. cDNA was purified using AMPure XP beads. Indexed libraries were generated and sequenced on the MGI DNBSEQ‐Tx platform with read lengths of 50 bp (Read 1) and 100 bp (Read 2).

### 5.9 Single‐cell RNA‐seq Data Processing and Cell Type Identification

Single‐cell RNA‐seq data were processed using Seurat (v4.4.0) [2]. Low‐quality cells with fewer than 600 detected genes were excluded, and genes expressed in fewer than three cells were removed. Data were log‐normalized and scaled, and the top 2,000 highly variable genes were identified for principal component analysis. Batch effects across samples were corrected using the Harmony algorithm. Cells were clustered based on the first 20 Harmony‐corrected principal components and visualized using UMAP and t‐SNE. Cell types were annotated using established marker genes.

### 5.10 snATAC‐seq Data Processing: Quality Control, Cell Clustering and Identification of Cell Types

Raw snATAC‐seq reads were processed using PISA (v1.1), aligned to the mouse mm10 genome with BWA, and further processed with bap2 to generate fragment files. Low‐quality nuclei were filtered using ArchR^3^ (v1.0.2) based on log10(unique fragments) ≥3 and TSS enrichment ≥4. Doublets were removed using the addDoubletScores function. Replicate consistency was evaluated by Pearson correlation analysis of PeakMatrix profiles.

### 5.11 Feature Plot Visualization

For snRNA‐seq: Generated feature plots using FeaturePlot() function in Seurat, which mapped gene expression levels onto UMAP embeddings; expression intensity was represented by a color gradient. For snATAC‐seq: Identified target chromatin regions. Visualized chromatin accessibility levels on UMAP embeddings using FeaturePlot() function in Signac, with accessibility intensity indicated by color gradient. All plots were adjusted for color scale and label size using ggplot2 package.

### 5.12 Spatial Stereo‐seq Data Analysis

Stereo‐seq data were processed as previously described [4]. Transcripts from 100 × 100 DNBs were aggregated into bin50 units, which served as the basic analytical unit. Bins outside tissue regions were manually excluded. Normalization, dimensionality reduction, and clustering were performed using Scanpy. Spatial clusters were annotated based on anatomical location, and gene module scores were calculated using sc.tl.score_genes.

### 5.13 Nissl Staining

Nissl staining was performed with the commercial Nissl Stain Kit in strict accordance with the manufacturer's recommended protocol. Mouse brain sections were incubated in methylene blue staining solution at 65°C for 10 min, followed by differentiation in Nissl differentiation solution for 3 min. Subsequently, the sections were immersed in ammonium molybdate solution for 5 min and immediately rinsed briefly with distilled water to prevent unintended decolorization. All stained sections were imaged under an inverted microscope (Leica, Germany).

### 5.14 SpaTrack Algorithm

The cell differentiation trajectory was inferred using the SpaTrack algorithm [5]. The starting region of the trajectory was manually defined based on the spatial coordinates of NSCs (X = 13000, Y = 10500), and 100 neighboring cells were selected as the initial cell population. Based on the spatial coordinates, the association strength matrix between cells (adata.obsp[‘trans’]) was calculated using optimal transport theory, quantifying the potential for cell state transition. Using the initial cells as the root node, the pseudotime value (adata.obs[‘ptime’]) for each cell was computed according to the intercellular transport relationships, reflecting the relative differentiation order of the cells along the inferred trajectory. Based on the spatial coordinates and pseudotime values, the rate and direction of cell state changes (adata.uns[‘V_grid’]) were estimated, and a velocity field (adata.uns[‘E_grid’]) was generated by interpolation on a grid, with the neighborhood size set to the 100 nearest neighboring cells. The sc.pl.embedding function was used to visualize the pseudotime and the cell differentiation direction.

### 5.15 Marker Genes of Clusters and GO Enrichment Analysis

Marker genes of different clusters were found by ‘FindMarkers’ of Seurat R package, with parameter (min.pct = 0.25, logfc.threshold = 0.25) and filtered by pvalue.adj<0.05. Gene set enrichment analysis, were processed with function ‘enrichGO’ using parameters (OrgDb = org. Mm.eg.db, pAdjustMethod = “BH”, pvalueCutoff = 0.01, qvalueCutoff = 0.05) of R package clusterProfiler (V3.16.1, https://bioconductor.org/packages/release/bioc/html/clusterProfiler.html).

### 5.16 Monocle2 Analysis

Monocle2 (v2.22.0) [6] was used to characterize the transition trajectories of NSCs. Briefly, three independent single‐cell clusters (C5_S2, C20, C0_C4) were integrated as a Seurat object and converted to a Monocle2 object using the newCellDataSet function. Standard preprocessing was performed: size factors were estimated (estimateSizeFactors) to normalize sequencing depth, gene expression dispersion was calculated (estimateDispersions), and genes expressed in ≥10 cells (min_expr = 0.1) were retained (detectGenes) for downstream analysis. For trajectory inference, preliminary cell clustering was performed to identify trajectory starting points. Dimensionality reduction was conducted via reduceDimension (method = ‘tSNE’, num_dim = 8), followed by cell partitioning into 8 clusters using clusterCells. Differential expression analysis (differentialGeneTest, grouping by cell clusters) identified genes with significant expression differences across clusters; the top 800 genes (smallest q‐values) were selected as ordering genes to represent dynamic expression during cell state transitions. Finally, trajectory construction was performed using reduceDimension (method = ‘DDRTree’), and cells were ordered along the trajectory (orderCells) to assign pseudotime values (defining relative positions along the developmental trajectory). Trajectory results were visualized with plot_cell_trajectory.

### 5.17 Gene Set Enrichment Analysis (GSEA)

GSEA was performed using the clusterProfiler R package to identify significantly enriched biological pathways in aNSCs vs. qNSCs. DEGs were ranked by their log2(fold change) values. We used the hallmark gene sets from the Molecular Signatures Database (MSigDB) as the reference gene set library. The enrichment significance was determined by 1,000 permutations, with pathways considered significantly enriched at a false discovery rate (FDR) < 0.25 and nominal *p*‐value < 0.05.

### 5.18 Cell Culture

NE‐4C, C17.2 and HEK293T cell lines were obtained from American Type Culture Collection (ATCC, USA). These cells were grown in DMEM medium (Vivacell, China) supplemented with 10% FBS and 1% penicillin/streptomycin at 37°C, 5% CO_2_. The primary NSCs were grown in NeuroCult Proliferation medium (STEMCELL, Canada).

### 5.19 Primary Culture of Mouse NSCs

Primary NSCs were isolated from the cerebral cortex of embryonic day 14.5 (E14.5) C57BL/6 mouse embryos. Briefly, embryonic cortical tissues were carefully microdissected under sterile conditions and immediately transferred into a small drop of pre‑chilled D‑Hank's balanced salt solution to maintain tissue viability. The harvested cortical tissues were thoroughly minced into fine pieces in a sterile 10 cm culture dish. For enzymatic dissociation, the minced tissue pellets were incubated in a digestion mixture containing trypsin (Biosharp, BL521A), 1% penicillin–streptomycin supplement (Biosharp, BL505A), and 250 U/mL DNase I (Beyotime, D7073). After brief vortexing, the samples were placed on a thermostable rotator and incubated at 37°C for 20 min to achieve sufficient tissue dissociation. Following digestion, the cell suspension was filtered through a 40 µm cell strainer, then centrifuged at 300 × g for 5 min at room temperature. The cell pellets were gently resuspended in fresh NeuroCult Proliferation Medium (STEMCELL Technologies, Canada) supplemented with 1% penicillin–streptomycin–glutamine. Single‐cell suspensions were obtained by gentle trituration approximately 20 times using a sterile pipette tip to avoid cell clumping. Subsequently, cells were incubated in a humidified incubator with 5% CO at 37°C for proliferation.

Typical neurospheres formed spontaneously within 3–4 days of culture. For routine passage, neurospheres were collected and dissociated with 0.125% trypsin at 37°C for 5 min. After enzymatic termination, cells were centrifuged at 300 × g for 5 min at room temperature, and resuspended in complete proliferation medium, and seeded for subsequent culture and experimentation.

### 5.20 Oxygen‐Glucose Deprivation and Reoxygenation (OGD/R)

To perform OGD/R on cultured NE‐4C, C17.2 and primary NSCs, the normal culture medium was replaced with Dulbecco's modified eagle's medium. The culture was then incubated in a hypoxia chamber aerated with 5% CO_2_, 94% N_2_ and 1% O_2_ at 37°C for 2 h. Then the cells were transferred back into the normal culture medium and incubated in normal culture conditions for 24 h.

### 5.21 qRT‐PCR

Total RNA was extracted from ipsilateral brain tissue using TriQuick Reagent. Reverse transcription was performed by HiFiScript All‐in‐one RT Master Mix following the manufacturer's instructions. qRT‐PCR was implemented in CFX (Bio‐rad) using MagicSYBR Mixture. The threshold cycle (Ct) values were used to quantify transcript levels. The relative expression level of each target gene was calculated using the 2^−ΔΔCT^ method. The primers used in this study are listed in Table S7.

### 5.22 Lentivirus and Adeno‐Associated Virus

NSCs were infected with lentivirus and selected with puromycin to establish stable cell lines overexpressing Nrsn1 (pLV3‐CMV‐CopGFP‐Puro‐Nrsn1‐3×FLAG), overexpress Smarcc1 (pCDNA3.1‐CMV‐Puro‐Smarcc1‐HA), knockdown Nrsn1 (pGreen‐EF1‐CopGFP‐Puro‐Nrsn1), or knockdown Smarcc1 (pGreen‐EF1‐CopGFP‐Puro‐Smarcc1) according to the manufacturer's instructions. The lentiviruses to overexpress Nrsn1 were purchased from OBiO Technology Co., Ltd. (Shanghai, China). Mice were infected with adeno‐associated virus (AAV) to stably overexpress Nrsn1 (pAAV‐Nestin‐Nrsn1‐HA‐P2A‐EGFP‐3xFLAG‐WPRE) in NSCs at the target injection site, following the manufacturer's instructions.

### 5.23 Transfection of Cells

One day before transduction, HEK‐293T, NE‐4C and C17.2 cells were seeded in 6‐well plates at a density of 3 × 10^5^ cells per well and incubated overnight. They were then infected with packaging plasmids and plasmids carrying target genes. Three days later, GFP expression was assessed in wells. For overexpression constructs, the negative control (NC) was generated by inserting a 350‐bp scrambled non‑coding, non‐targeting DNA sequence with the same length as the Nrsn1 coding fragment. For knockdown constructs, the NC consists of a scrambled non‐targeting sequence.

### 5.24 shRNA Transfection

shRNA knockdown was performed using adenovirus transduction as described previously. shRNA targeting mouse Nrsn1 was synthesized by Tsingke (Beijing, China). The shRNA1 targeting sequence for Nrsn1 is 5′‐GCCAAGAGCTATTCCAAAGAA ‐3′. The shRNA2 targeting sequence for Nrsn1 is 5′‐GAGCGAATCGCAGACATTAAA ‐3′. Two shRNA sequences were cloned into the shuttle vector pLKO‐ EGFP and packaged in 293T cells to harvest high‐titer recombinant adenovirus. For knockdown studies, cells were seeded at a density of 5  ×  10^5^ cells per well in 6‐well plates. The next day, cells were infected with NC, shRNA1 and shRNA2 adenovirus. Infection efficiency was determined by counting the number of GFP‐positive cells under a fluorescence microscope.

### 5.25 Western Blot Analysis

Total protein was extracted from cells using RIPA lysis buffer with protease inhibitor PMSF. Protein concentration was determined by the BCA assay. Protein samples (10 µg) were subjected to electrophoresis on 12% SDS‐PAGE gels and then transferred to polyvinylidene fluoride membranes (PVDF, Immobilon, America). The membranes were incubated with primary antibodies including anti‐Nrsn1 (1:1000, Invitrogen, PA5‐98607); anti‐Tuj1 (1:1000, abcam, ab18207); anti‐β‐actin (1:5000, Proteintech, 66009‐1‐Ig); anti‐Foxa2 (1:1000, CST, 8186) and anti‐Smarcc1 (1:1000, abcam, ab305037) overnight at 4°C. The membranes were next incubated with secondary antibodies for 1 h at room temperature (1:5000, Proteintech) and were detected using the ECL Enhanced Kit. The original immunoblot bands are shown in Figure S11.

### 5.26 TF Regulation Activity Prediction

To identify cell type‐specific chromatin open regions, peak calling was performed using ArchR. First, the addGroupCoveragesfunction was used to create pseudo‐bulk replicates for each cell group defined by “Clusters”, thereby merging signals from similar cells to provide sufficient sequencing depth for subsequent analysis. Reproducible peak sets were then called using the addReproduciblePeakSetfunction (grouped by cluster, using the MACS2 algorithm). Subsequently, motif annotation information was added using addMotifAnnotations, and the getMarkerFeaturesfunction combined with peakAnnoEnrichmentwas used to analyze transcription factor motifs significantly enriched in each cluster (cutOff: FDR ≤ 0.1 & Log2FC ≥ 0.5).

### 5.27 Dual‐Luciferase Reporter Assays

The pGL3‐Nrsn1 reporter plasmid was constructed by inserting the promoter region of the mouse Nrsn1 gene (2000 bp upstream of the transcription start site) into the multiple cloning site (MCS) of the pGL3‐basic vector. The pcDNA3‐Foxa2 overexpression plasmid was generated by cloning the full‐length coding sequence of mouse Foxa2 into the pcDNA3‐basic vector (Miaoling Biotech), and the empty pcDNA3‐basic vector was used as a negative control. The pRL‐TK vector (Promega), which expresses Renilla luciferase under the control of the thymidine kinase (TK) promoter, was used as an internal reference to normalize the transfection efficiency. For transfection, cells were seeded into 24‐well plates at a density of 3 × 10^5^ cells per well and cultured for 24 h until they reached 70%−80% confluence. Transfection was performed using Polyethylenimine linear according to the manufacturer's instructions. At 48 h post‐transfection, the cells were lysed and centrifuged at 12,000 × g for 5 min at 4°C to remove insoluble debris. The luciferase activity was measured using the Dual‐Lumi II Dual‐Luciferase Reporter Gene Assay Kit by the microplate reader. The relative luciferase activity (RLA) was calculated as the ratio of firefly luciferase activity to Renilla luciferase activity.

### 5.28 ChIP‐qPCR

Following the ChIP Assay Kit (Beyotime, P2080S) instructions, C17.2 cells were treated with formaldehyde and glycine solution. Chromatin was fragmented by sonication. Anti‐Foxa2 (CST, 8186) and anti‐IgG (CST, 3900) were coupled to magnetic beads overnight at 4°C. For immunoprecipitation, a 100 µl aliquot of sonicated chromatin was incubated overnight with antibody‐coupled magnetic beads, washed, and eluted by incubation at 65°C for 1 h with brief vortexing every 15 min. To completely revert crosslinking, the eluted chromatin was incubated overnight at 65°C with proteinase K (Beyotime, ST537). The fragmented chromatin was purified using a ChIP DNA Clean and Concentrator Kit (Zymo Research).

### 5.29 Immunoprecipitation

Cell lysates from 5 × 10^6^ cells were prepared using the IP buffer supplemented with 2 mM PMSF. HA‐Nanoab‐MagneticBeads (LABLEAD, HNM‐50‐2000) or Flag‐Nanoab‐Magnetic beads (LABLEAD, FNM‐25‐1000) were incubated with the lysates on a rotator at 4°C for 6 h. Beads were collected and stored at −80°C until further use.

### 5.30 LC‐MS

LC‐MS analysis was performed by PTM BIO (China). Mouse bead samples were lysed in 100 mM ammonium bicarbonate. A two‑step on‑bead tryptic digestion strategy was applied for protein digestion. Briefly, proteins were first digested directly on beads with trypsin at a final concentration of 20 ng/µL overnight. Subsequently, samples were reduced with 5 mM DTT at 37°C for 60 min and alkylated with 11 mM IAA at room temperature in the dark for 45 min. To achieve sufficient digestion of protein regions masked by disulfide bonds, a secondary digestion was performed with 10 ng/µL trypsin for an additional 4 h. The resulting peptides were acidified and desalted, and no HPLC fractionation was performed prior to LC‐MS analysis.

Peptides were separated on an EASY‐nLC 1200 UPLC system with a 30 min linear gradient (6%–80% acetonitrile with 0.1% formic acid) at 700 nL/min. Mass spectrometry was performed on an Orbitrap Exploris 480 equipped with a nano‐spray ionization (NSI) source (2300 V; FAIMS CV = −45 V). Data were acquired in data‐independent acquisition (DIA) mode. Full MS (m/z 450–850) and MS/MS spectra were both recorded at 30,000 resolution; HCD fragmentation energy was set to 28%, and AGC target was 3 × 10^6^.

Raw data were analyzed using DIA‐NN v1.8. Spectra were searched against the Mus_musculus_10090_SP_20241202.fasta database (17,236 sequences) plus a decoy database. Trypsin/P was set as the protease with maximum 1 missed cleavage. Fixed modifications included N‐terminal methionine excision and cysteine carbamidomethylation. Precursor‐level q‐values were controlled at ≤0.01, and protein‐level identifications were further filtered using Global.PG.Q.Value ≤0.01. Proteins supported by at least one unique peptide were retained for the primary interactome dataset. For sample cohorts with biological replicates, shared and unique proteins across replicates were separately analyzed; no uniform quantitative cutoff for replicate protein detection was applied.

For sensitivity analysis, an additional filtering criterion of at least two unique peptides was applied to the primary interactome dataset. Fisher's exact test was used to discriminate true interactors from non‐specific background binding and perform functional enrichment analysis. Functional terms with fold enrichment > 1.5 and *p* value < 0.05 were defined as significantly enriched entries, and high‐confidence interacting proteins were retained after background filtering based on statistical results. The primary interactome dataset consisted of proteins exclusively identified in the OE‐Nrsn1 pulldown (absent from the NC group), passing Global.PG.Q.Value ≤0.01 and supported by at least one unique peptide. Proteins supported by at least two unique peptides were additionally examined in the sensitivity analysis. Protein‐protein interaction networks were built via the STRING database (confidence score > 0.7) and visualized using the R package visNetwork.

### 5.31 Nuclear and Cytoplasmic Protein Extraction Protocol

Nuclear and cytoplasmic proteins were extracted using the Nuclear and Cytoplasmic Protein Extraction Kit following the manufacturer's standard protocol with minor modifications. Cells were washed twice with PBS and then were scraped off the culture dish with a cell scraper. Cytoplasmic Extraction Buffer A containing 1 mM PMSF was added. The centrifuge tube was placed on ice, and then Cytoplasmic Buffer B was added. The mixture was incubated on ice, followed by centrifugation at 12,000 × *g* for 5 min at 4°C to get cytoplasmic protein fraction. The remaining pellet after cytoplasmic protein collection was resuspended with Nuclear Extraction Buffer containing 1 mM PMSF. The centrifuge tube was placed on ice, and the pellet was vortexed for 15 s every 30s, with a total incubation time of 30 min. After incubation, the mixture was centrifuged at 12,000 × *g* for 10 min at 4°C. The supernatant (nuclear protein fraction) was transferred.

### 5.32 Stereotaxic Surgeries and Injection

Mice (approximately 5 or 9 weeks old) were anesthetized with 2% isoflurane and placed in a mouse stereotaxic instrument (RWD, China). The skull was drilled to make a burr hole above the lateral ventricle (AP+0, ML‐1, DV‐2.25 mm) for AAV and NSCs injection. A total of 5 × 10^5^ NSCs, suspended at a density of 1 × 10^5^ cells/µL, were bilaterally stereotactically injected into the subventricular zone using a pressure microinjector (Hamilton 701RN) at a slow infusion rate of 1 µL/min. For the PBS group, the equal volume of PBS was injected under the identical stereotactic conditions [7]. For AAV injection, we injected 3 ul of virus (AAV‐OE‐Nrsn1, 3.84 × 10^12^ vg/ml; AAV‐NC, 3.30 × 10^12^ vg/ml) to 5‐week‐old mice. The injection needle was withdrawn 5 min after the infusion (Hamilton 701RN) at a slow rate of 1 µL/min. EdU injections were performed daily after MCAO/R. All animals were intraperitoneally injected with 200 mg/kg EdU. Mice were sacrificed at 7 or 14 days after MCAO/R, and tissue fixation was performed prior to cell proliferation analysis using the BeyoClick EdU‐594 Cell Proliferation Detection Kit.

### 5.33 Motor Function Assessment

Balance beam, ladder rung walking, and rotarod tests were performed preoperatively and at 1–4 weeks postoperatively following established procedures [8]. Investigators were blinded to the treatment groups during all tests.

### 5.34 MRI

Mice were anesthetized and scanned by MRI (United Imaging, America) to detect the infarct area in the ipsilateral brain. Mice were imaged with a T2‐weighted fast spin echo sequence using on the 3T MRI scanner.

### 5.35 Golgi Staining and Analysis

Golgi staining was conducted using the FD rapid Golgi Stain kit according to the manufacturer's instructions. Mice were anesthetized and sacrificed, and the brains were removed quickly and immersed in the mixture of Solution A and B for 2 weeks at room temperature in the dark. The brain was then transferred into Solution C for 48 h at 4°C. Sections were cut with 100 µm thickness using a vibratome (Lecia, Germany) and stained with D and E mixture. Images were captured by an inverted microscope using Z‐stack images (Lecia, Germany). Golgi‐stained neurons were reconstructed using Fiji‐Image J. The total dendritic length, the number of dendritic spines and intersections were quantified and analyzed by Sholl analysis according to the previous study [9].

### 5.36 Optical Density Analysis

ImageJ software was used to analyze the average optical density (AOD) (integrated optical density/area) to indicate the level of protein expression. Three different visual immunofluorescence fields were acquired from different tissue sections, and the average AOD was calculated. All tissue sections were stained in the same batch, and images were acquired with the same imaging threshold and exposure time to ensure consistency in image analysis.

### 5.37 Protein Stability and Proteasome Inhibition Assays

CHX chase assays and MG132 proteasome inhibition assays were performed in C17.2 cells. For CHX chase experiments, cells were treated with 100 µM cycloheximide (MCE; HY‐12320) for 0, 4, and 8 h to block de novo protein synthesis. Cells were harvested at the indicated time points, and endogenous Smarcc1 protein levels were examined by WB. For proteasome inhibition assays, cells were incubated with 10 µM MG132 (MCE; HY‐13259G) for 0, 12, 24, and 48 h to suppress 26S proteasome‐mediated protein degradation. Total protein was extracted for WB.

### 5.38 Statistics Analysis

All data are presented as mean ± SEM. Statistical analyses were performed using GraphPad Prism 10. Two‐tailed unpaired Student's t tests or one‐way ANOVA with Tukey's post hoc test were applied as appropriate. Statistical significance was set at *P*   < 0.05, and expressed as *P  < 0.05, **P < 0.01, ***P < 0.001, and ns means not significant.

## Author Contributions

Z.D. and C.L. conceived and designed the study. R.Z. performed the majority of the experiments and analyzed data. R.Z. and Z.D. wrote and revised the manuscript. C.L., Z.Y., K.Z. and R.Z. conducted the analysis of the snRNA‐seq, snATAC‐seq and Stereo‐seq data. R.Z. Y.W, B.C. and Z.Y. performed cellular assays, including Western blot, Co‐IP and qPCR etc. R.Z. and M.L. established the MCAO/R mouse model, followed by Nissl, TTC and Golgi staining. Z.Y, Z.L., Y.X. and Y.W completed the behavioral tests and immunofluorescence staining. Z.Y. performed MRI measurements. P.Z., K.S., W.B., M.Z, J.Q., J.C. and X.Y. participated in the discussion. All authors read and commented on the draft version of the manuscript and approved the final version.

## Conflicts of Interest

The authors declare no conflicts of interests.

## Supporting information




**Supporting File 1**: advs77547‐sup‐0001‐SuppMat.docx.

**Supporting File 2**: advs77547‐sup‐0002‐SuppMat.xlsx.

## Data Availability

The data that support the findings of this study are available in the supplementary material of this article. The snRNA‐seq, snATAC‐seq and Stereo‐seq datasets generated in this study have been deposited at the GEO repository. Accession numbers: GSE332910; https://www.ncbi.nlm.nih.gov/geo/query/acc.cgi?acc=GSE332910. They are also deposited at the China National Center for Bioinformation (CNCB). Accession numbers: OMIX014345; https://ngdc.cncb.ac.cn/omix/preview/iTKW0LED. They are also deposited at figshare: https://figshare.com/s/10e8c6191b9a013d97d2; https://figshare.com/s/4c17c8a75d899e080347. The code and markdown scripts used to do the analysis in the paper have been deposited at figshare: https://figshare.com/s/4a4443e586d9151ad515. The original immunofluorescence image has been uploaded at figshare: https://figshare.com/s/af36a59aa6731ba67b3a.
