# Supplementary material for: Nrsn1–Smarcc1 Coupling Regulates Neural Stem Cell Differentiation and Chronic‐phase Recovery After Ischemic Stroke
Source: Adv Sci (Weinh). 2026 Sep 3:e77547. Online ahead of print. doi: 10.1002/advs.77547 (PMC13542395; doi:10.1002/advs.77547)
Supplement: Supplementary file 1 [file ADVS-9999-e77547-s002.docx]

**Supplementary figure 1. Differentiation of NSCs in the striatum post-ischemic stroke**

(A) Representative Nissl staining image of the mouse brain (left panel); red and blue boxes indicate the ROIs corresponding to the dorsal striatum and ventral striatum, respectively. Corresponding IF images of Nestin and Tuj1 in the dorsal striatal ROI (red-boxed region; middle panel) and ventral striatal ROI (blue-boxed region; right panel) at different time points post-stroke. Scale bar: 200 μm. (B) Feature plot showing the representative marker genes of different cell types in snRNA-seq and snATAC-seq. (C) Circle plots displaying the ligand-receptor interactions in NSC, astrocyte and endothelial, and the edge width represents communication probability. (D) Percentage of NSCs number post-stroke in the striatum. (E) Heatmaps of marker genes of NSCs in different time points post-stroke in the striatum.

**Supplementary figure 2. Nrsn1 acts as a critical regulator governing neuronal differentiation of NSCs**

(A) t-SNE visualization of NSC subclusters (left). Feature plots showing the expression distribution of marker genes *Pax6*, *Nrxn3* and *Ccnd2* across NSCs subclusters (right). (B) Dynamic changes in the percentage of qNSCs and aNSCs populations post-stroke, demonstrating a progressive shift from quiescent to activated NSC states. (C) GSVA heatmap displaying the enrichment scores of hallmark pathways across different cell types. (D) Pseudotemporal trajectory analysis of NSC subclusters. The left panel shows the distribution of qNSCs (blue), aNSCs 1 (red), and aNSCs 2 (green) along the differentiation trajectory. The right panel is the pseudotime progression, with cells colored from early (light blue) to late (dark blue). (E) Pseudotemporal trajectory analysis of NSCs, APCs, and astrocytes. The left panel shows the distribution of NSCs (blue), APCs (green), and astrocytes (red) along the differentiation trajectory. The right panel displays the pseudotime progression, with cells colored by pseudotime value from early (light blue) to late (dark blue). (F) Volcano plot showing DEGs between APC and NPC (left). Genes upregulated in NPC are marked in red (n = 202), downregulated in blue (n = 274). Key genes are labeled. Top enriched GO biological processes for NPC-upregulated genes (right). (G) The relative mRNA expression of *Nrsn1* in different time points post-OGD/R were measured by RT-qPCR, *n*= 4. Data are presented as the mean value ± SEM. ***P*  < 0.01.

**Supplementary figure 3. Nrsn1 regulates the differentiation of NSCs in vitro**

(A) RT-qPCR analysis of relative mRNA expression of *Nrsn1* in different groups of NE-4C (left) and C17.2 (right) cells, *n*= 3. (B) WB results show the expression of Nrsn1, GAPDH and β-actin in different groups of NE-4C (upper) and C17.2 (lower). (C) Densitometric quantification of the WB bands shown in (B), normalized to β-actin expression, n= 3. (D) RT-qPCR analysis of relative mRNA expression of *Nrsn1*, *Nes*, *Pax6* and *Tubb3* in different groups of C17.2 cells, *n*= 3. (E) RT-qPCR (left) and WB (middle) analysis of relative mRNA expression of Nrsn1 in different groups of sh1-Nrsn1-NE-4C cells, and densitometric quantification of the WB bands (right), normalized to β-actin expression, *n*= 3. (F) RT-qPCR analysis of relative mRNA expression of *Nrsn1* in different groups of sh1-Nrsn1-C17.2 (left) and sh2-Nrsn1-C17.2 (right) cells, *n*= 3. (G) IF of GFP and Tuj1 in different groups of NE-4C cells. Scale bar: 20 μm. (H) Percentage of Tuj1^+^ and GFP^+^ cells among total GFP^+^ cells, *n*= 12 images per group. Data are presented as the mean value ± SEM. **P*  <  0.05, ** *P*  <  0.01, ****P*  <  0.001, and ns means not significant.

**Supplementary figure 4. The presence of Nrsn1 transcriptionally regulated by Foxa2 mediates the neuronal differentiation of NSCs after OGD/R**

(A) The *Foxa2*-binding motif was predicted by [JASPAR](https://jaspar.elixir.no/) , and the potential *Foxa2* binding sites in the *Nrsn1* promoter region are shown. (B) ChIP-qPCR analysis validates direct Foxa2 binding to the Nrsn1 promoter. ChIP-qPCR was performed using anti-Foxa2 antibody and IgG control on chromatin extracted from experimental cells. Three candidate FOXA2 binding regions (Region 1, Region 2 and Region 3, the sequence was shown in Table S8, Supporting Information) of the Nrsn1 promoter were amplified. (C) RT-qPCR analysis of relative mRNA expression of *Foxa2* in different groups, n= 4. Data are presented as the mean value ± SEM. * P  <  0.05.

**Supplementary figure 5. Nrsn1 interacts with Smarcc1 and facilitates its nuclear translocation to promote neural stem cell differentiation after OGD/R**

(A) Silver-stained SDS-PAGE gel showing protein quality assessment. (B) co-IP assays were performed in NSCs transfected with Flag-tagged Nrsn1 overexpression plasmid (OE-Nrsn1) or NC. (C) GO analysis of differentially expressed proteins (332 Nrsn1-binding proteins) in the OE group with respect to molecular function (MF). (D) GO analysis of 68 interacting proteins with respect to biological processes (BP) and molecular function (MF, right). (E) RT-qPCR analysis of relative mRNA expression of *Smarcc1*, n= 3. (F) RT-qPCR analysis of *Nrsn1* and *Smarcc1* expression, *n*= 3. (G) WB and densitometric quantification show the expression of Smarcc1, Nrsn1 and β-actin, *n*= 3. (H) WB and densitometric quantification show the expression of Nrsn1, Smarcc1 and β-actin, *n*= 3. (I) IF of Smarcc1 in C17.2 cells under Ctrl and OGD/R conditions. The arrow indicates Smarcc1 in the cytoplasm. Data are presented as the mean value ± SEM. **P*  <  0.05, ****P*  <  0.001, and ns means not significant.

**Supplementary figure 6. Nrsn1 overexpression promotes NSC differentiation into neurons in the brains post-stroke.**

(A) IF of Nestin, GFP and Nrsn1 in the SVZ at 3 w after AAV injection. (B) IF of GFP and DCX in different groups at 2 w post-stroke. (C) Percentage of GFP^+^ and DCX^+^ NSCs among total GFP^+^ NSCs (left), and percentage of GFP^+^ and Tuj1^+^ NSCs among total GFP^+^ NSCs (right) of 2 w post-stroke in mice, *n* =  9 images per group. (D) IF of GFP, Tuj1 and DCX in different groups at 4 w post-stroke. (E) Percentage of GFP^+^ and DCX^+^ NSCs among total GFP^+^ NSCs (left), and percentage of GFP^+^ and Tuj1^+^ NSCs among total GFP^+^ NSCs (right) of 4 w after stroke in mice, *n* =  9 images per group. Scale bar: 50 μm (A, B and D). Data are presented as the mean value ± SEM. **P*  <  0.05, ****P*  <  0.001, and ns means not significant.

**Supplementary figure 7. Therapeutic effects of Nrsn1-Overexpressing NSCs transplantation in MCAO/R**

(A) Nissl staining of infarct area in the brain at 2 w and 4 w after treatment. Scale bar: 200 μm. (B) IF of GFP and GFAP in different groups at 4 w post-stroke. Scale bar: 50 μm. (C) Percentage of GFP^+^ and GFAP^+^ NSCs among total GFP^+^ NSCs of 4 w after stroke in mice, *n* =  9 images per group. Data are presented as the mean value ± SEM. **P* <  0.05.

**Supplementary figure 8**. **Schematic diagram of the mechanism underlying Nrsn1-mediated regulation of NSC differentiation into neurons**

After stroke, Foxa2 regulates the transcription of Nrsn1, and then Nrsn1 interacts with Smarcc1 and is translocated into the nucleus, where it modulates the differentiation of NSCs into neurons via the BAF complex, thus enhancing motor and neurological functional recovery in ischemic stroke model mice.

**Supplementary figure 9. Prediction of nuclear localization and export sequences of Nrsn1**

(A) and (B) The scores and sequence of predicted bipartite Nuclear Localization. (C) The scores and sequence of predicted bipartite Nuclear Localization.

**Supplementary figure 10. CHX and MG132 inhibitor assays exclude regulation of Smarcc1 protein stability and ubiquitin-proteasome degradation by Nrsn1.**

(A) CHX chase immunoblot showing Smarcc1 decay rates in NC and Nrsn1-OE cells at 0, 4, 8 h post CHX treatment. Quantified Smarcc1 signals normalized to β-actin are shown below blots. (B) MG132 treatment immunoblot measuring proteasome-dependent Smarcc1 accumulation in NC and Nrsn1-OE cells at 0, 12, 24, 48 h. β-actin was used as loading control.

**Supplementary figure 11. Original immunoblotting bands.**
